# ChCpc1, a bZIP transcription factor, coordinates amino acid synthesis and autophagy and modulates conidiation and virulence in *Cochliobolus heterostrophus*

**DOI:** 10.1128/mbio.00845-25

**Published:** 2025-07-21

**Authors:** Huilin Yu, Jiyue Zhang, Longhao Su, Mengjiao Jia, Yuanyuan Tian, Hongyu Pan, Xianghui Zhang

**Affiliations:** 1 College of Plant Science, Jilin University 12510, Changchun, China; Cornell University, Ithaca, New York, USA

**Keywords:** amino acid synthesis, autophagy, conidiation, Cpc1, stress response, transcription factor, virulence

## Abstract

*Cochliobolus heterostrophus*, a fungal plant pathogen, is the causal agent of southern corn leaf blight. The bZIP transcription factor Cpc1 is required for response to oxidative stress and amino acid starvation in the saprobe, *Neurospora crassa*, and the human pathogen, *Candida albicans*. However, although conserved in fungal plant pathogens, Cpc1 function and regulatory mechanisms are still largely unknown. Through targeted gene deletion, we demonstrated that *ChCPC1* was necessary for nitrogen assimilation and response to amino acid starvation during conidiation and the infection process. In addition, amino acid synthesis and autophagy induction were also impaired in Δ*Chcpc1* mutants. We found that ChCpc1 binds to the promoter region of *ChARG1* and *ChARG4* to activate arginine synthesis to counteract amino acid depletion. Amino acid deprivation induced autophagy in *C. heterostrophus*, and it depended on the direct activation of *ChATG* genes by ChCpc1, which confirmed the genetic relationship between ChCpc1 and *ChATG* genes. Additionally, we demonstrated that ChCpc1 forms a heterodimer with another bZIP transcription factor, ChAtf1, to enhance the binding of ChCpc1 to the promoter regions of *ChARG4* and *ChATG8*. Furthermore, mitogen-activated protein kinase ChChk1 phosphorylated ChCpc1 to counteract amino acid depletion in *C. heterostrophus*, and the other two components of the MAPK pathway (ChSte7 and ChSte11) were also involved in the response to amino acid starvation stress, conidiation, and virulence in *C. heterostrophus*. This study confirmed that ChCpc1 coordinates amino acid synthesis and autophagy to modulate conidiation and virulence in *C. heterostrophus*.

**IMPORTANCE:**

Southern corn leaf blight (SCLB) caused by *Cochliobolus heterostrophus* is a destructive disease that threatens global maize (*Zea mays*) production. The bZIP transcription factor Cpc1 is conserved in fungal plant pathogens; however, the biological function and the regulatory mechanism are still largely unknown. In this study, we characterized the function of ChCpc1 in *C. heterostrophus*, especially noting that ChCpc1 was found to be involved in response to amino acid starvation and autophagy. Additionally, we revealed that ChCpc1 directly targets arginine synthesis genes and autophagy-related genes to counteract amino acid depletion during the infection process of *C. heterostrophus*. Importantly, we found that another bZIP transcription factor, ChAtf1, and protein kinase ChChk1 were also important for responding to amino acid depletion functioning upstream of ChCpc1. The discoveries could broaden the understanding of how plant pathogenic fungi respond to nutrient stress to ensure successful infection.

## INTRODUCTION

Microorganisms live in a complex environment, and changes in environmental conditions will affect the growth and development of microorganisms dramatically. In particular, plant pathogenic fungi suffer from the host’s defense response during the infection process, and the pathogens themselves must overcome various unfavorable conditions to effect successful infection (1, 2). Especially, unlike animals, microbes and plants have difficulty migrating to more suitable environments when faced with adverse conditions, so they must evolve appropriate regulatory mechanisms to adapt to adverse environmental conditions (3, 4). Many studies have shown that plant pathogenic fungi will encounter several adverse factors during the infection process, such as reactive oxygen species (ROS), reactive nitrogen species (RNS), and nutrient deficiency (2–4).

ROS burst is a common weapon of plants when they are faced with a series of biotic stresses, in particular in the context of defense against pathogens (5). In *Sclerotinia sclerotiorum*, the SWI/SNF complex and its co-operator co-regulate the transcription of *hsp* genes and antioxidant enzyme genes to respond to ROS produced by the host (6). RNS derived from nitric oxide displayed antimicrobial activities to pathogens by reacting with microbial cellular components. In *Fusarium graminearum*, the transcription factor FgAreB recruits the chromatin-remodeling complex SWI/SNF to promote the transcription of genes involved in nitrosative stress (7). Nutrient acquisition during the infection process and the synthesis of primary metabolite components, such as amino acids, are also important to produce full virulence in pathogenic fungi (2, 8–10). However, how plant pathogenic fungi integrate multiple pathways in response to nutrient stress to promote conidiation and infection has been explored seldomly.

Amino acid is an important organic compound that plays an essential role in cell proliferation, protein synthesis, and energy production in microbes. In addition, the amino acid synthesis-related genes are not only involved in amino acid biosynthesis but also essential for mycelial growth, conidiation, and pathogenicity in plant fungal pathogens. For example, *MoIVD*-mediated leucine catabolism is required for vegetative growth, conidiation, and full virulence of the rice blast fungus, *Magnaporthe oryzae* (11). The isopropylmalate isomerase MoLeu1 orchestrates leucine biosynthesis, fungal development, and pathogenicity in *M. oryzae* (12), and *MoARGs*, which are involved in arginine biosynthesis, are essential for growth, conidiogenesis, sexual reproduction, and pathogenicity in *M. oryzae* (9). Acetolactate synthases regulatory subunit and catalytic subunit genes *VdILVs* are involved in branched chain amino acid biosynthesis, microsclerotial and conidial formation, and virulence in *Verticillium dahlia* (13).

Autophagy is a degradative pathway that is responsible for degradation and recycling of organelles and macromolecules, and it is important for development, reproduction, and virulence in fungi. In previous studies, many *ATG* genes were identified, and the regulatory pathways were investigated (10, 14, 15). In *C. heterostrophus*, Δ*Chatg4* and Δ*Chatg8* mutants showed significant changes in vegetative growth, asexual development, sexual development, autophagy, and virulence. In addition, deletion of *ChATG4* and *ChATG8* disordered Cdc10 subcellular localization and formation of septin rings (14). The serine/threonine protein kinase Rim15 promoted biotrophic growth by coordinating cycles of autophagy and glutaminolysis in invasive hyphae in *M. oryzae* (10). Corral-Ramos et al. (15) found that autophagy mediated nuclear degradation after hyphal fusion and functioned in the control of nuclear distribution in *Fusarium oxysporum* (15). MoAtg4 phosphorylation mediates the coordination between autophagy and CWI signaling, which is the basis for the development and pathogenicity of *M. oryzae* (16, 17).

Basic leucine zipper (bZIP) transcription factors (TFs) are major regulatory elements in development and virulence in fungi. However, only a few bZIP TFs have been identified functionally in pathogenic fungi, and the regulatory networks have been investigated poorly. The bZIP transcription factor BIP1 of the rice blast fungus was essential for infection and critical for the expression of early invasion-related genes in appressoria (18). Another bZIP transcription factor, Atf1, is essential for full virulence, deoxynivalenol production, and stress tolerance in *F. graminearum* (19). VdAtf1 controlled pathogenesis through the regulation of nitric oxide (NO) resistance and inorganic nitrogen metabolism rather than oxidative resistance, and it was important for penetration peg formation in *V. dahliae* (20). Cpc1 is a homolog of the bZIP transcription factor Gcn4 in yeast, which was involved in the expression regulation of amino acid biosynthesis genes in *Candida albicans*, *Verticillium longisporum*, and *Fusarium fujikuroi* (21–23). However, how the Cpc1 coordinated amino acid synthesis to promote conidiation and virulence was not revealed in phytopathogenic fungi.

In this study, the connection between transcription factor ChCpc1 and amino acid synthesis or autophagy was investigated in *C. heterostrophus*, which is a destructive pathogen of corn (*Zea mays*). Our results demonstrated that transcription factor ChCpc1 coordinated *ChARGs* and *ChATGs* to regulate conidiation and virulence under amino acid starvation in *C. heterostrophus*. In addition, another bZIP transcription factor, ChAtf1, promotes the binding of ChCpc1 at the promoters of *ChARG4* and *ChATG8*. Moreover, ChCpc1 is a target of the mitogen-activated protein kinase ChChk1. When considered together, our study revealed the comprehensive regulatory network of ChCpc1 in conidiation and virulence in *C. heterostrophus*.

## RESULTS

### Nitrogen and amino acid metabolism involved in conidiation and the infection process of *C. heterostrophus*

In our previous study, the infection-specific transcriptional patterns of *C. heterostrophus* were uncovered, and several genes involved in asexual development and virulence were enriched (24). What was more notable was that many nitrogen and amino acid metabolism pathways were enriched on 12 or 24 hpi, which included valine, leucine, and isoleucine degradation, cysteine and methionine metabolism, sphingolipid metabolism, arginine and proline metabolism, tyrosine metabolism, and phenylalanine metabolism (Fig. S1A) (24). Some studies revealed that genes for amino acid synthesis were necessary for growth, conidiation, sexual reproduction, and pathogenicity in plant pathogenic fungi (4, 9, 11, 12). However, how those genes were regulated and how they were coordinated during conidiation and the infection process in plant pathogenic fungi are seldom reported.

*ScGCN4* and *CaGCN4*, which are the homologous genes of *ChCPC1* in *Saccharomyces cerevisiae* and *Candida albicans*, act as a global regulator that coordinated both metabolic and morphogenetic responses to amino acid starvation (21). In addition, *ChCPC1* was significantly induced in conidia and during the infection process (Fig. S1B). To further elaborate on the role of *ChCPC1*, we sought to better understand how *ChCPC1* coordinated nitrogen assimilation and amino acid synthesis during *C. heterostrophus* conidiation and infection. *ChCPC1* was predicted to encode a 446-amino acid protein that included a bZIP_GCN4 domain. Phylogenetic analysis indicated that the Cpc1 protein is conserved in yeast and filamentous fungi, and ChCpc1 shares a higher identity with the orthologs of *Cercospora zeae-maydis*, *Alternaria alternata*, and *Setosphaeria turcica*, which all belong to Dothideomycetes (Fig. S1C).

### The bZIP transcriptional factor ChCpc1 was required for conidiation, melanin production, and virulence in *C. heterostrophus*

**Fig 1:**
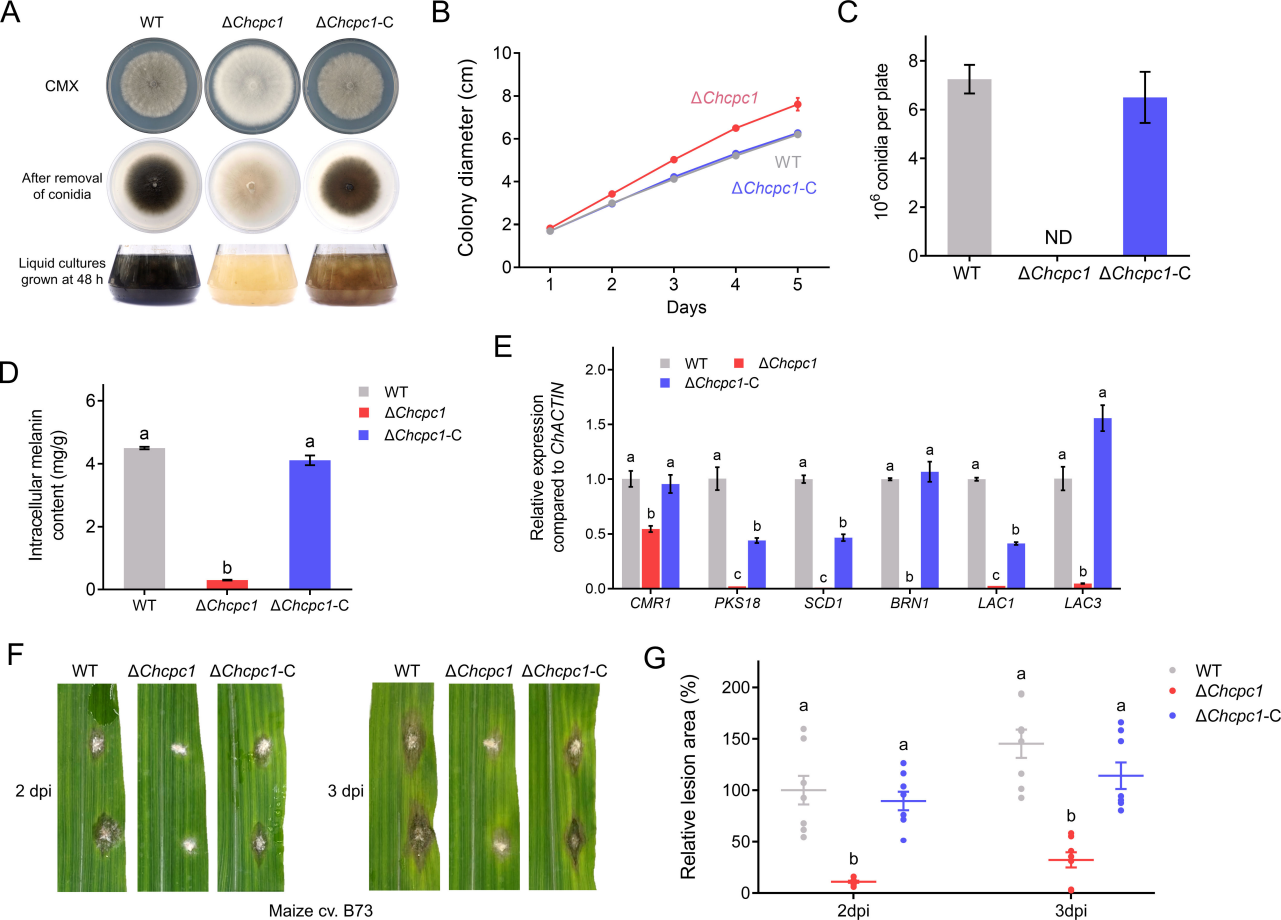
ChCpc1 was required for conidiation, melanin production, and virulence in *C. heterostrophus*. (**A**) Morphology of indicated strains on CMX agar plates and liquid cultures. (**B**) Statistical analysis of the colony diameter of indicated strains grown on CMX for 5 days. (**C**) Conidiation of indicated strains. (**D**) Statistical analysis of the intracellular melanin content of indicated strains. (**E**) Relative expression levels of melanin biosynthesis-related genes in WT, Δ*cpc1* mutant, and complemented strains. (**F**) Virulence of WT, Δ*cpc1* mutant, and complemented strains on detached maize leaves at 2 and 3 days post-inoculation (dpi). (**G**) Statistical analysis of the lesion area of indicated strains at 2 and 3 dpi. GraphPad Prism program’s *t-*tests and multiple *t-*tests were used for the analysis of significant differences. The bars indicate the standard error of means of three replications.

The coding region of *C. heterostrophus ChCPC1* in wild-type (WT) C4 was replaced by the *HPH* gene conferring hygromycin resistance using homologous gene recombination. Compared with the WT strain, Δ*cpc1* deletion strains showed marginally faster mycelial growth, but the colonies of deletion strains were completely white (Fig. 1A and B). Because conidia are the main source of the dark gray/green pigmentation of WT, the white coloration of mutant mycelia reflected low production of conidia. To evaluate the conidiation of the mutants, 9-day-old colonies grown on CMX were harvested. Δ*cpc1* deletion strains were unable to produce conidia, whereas the WT and complemented strains produced more than 6 × 10^6^ conidia/plate (Fig. 1C). This indicated that the deletion of *ChCPC1* did not affect the mycelial growth of *C. heterostrophus*; however, ChCpc1 was necessary for conidiation.

For the white colony of the Δ*cpc1* mutant strains, we asked whether *ChCPC1* deletion impacted melanin production. After 48 h of cultivation, the melanin accumulation of WT, Δ*cpc1* mutant strain, and complemented strain was determined. The melanin production of the Δ*cpc1* mutant strain was significantly reduced (Fig. 1D). In addition, six melanin biosynthesis-related genes (*CMR1*, *PKS18*, *SCD1*, *BRN1*, *LAC1*, and *LAC3*) were downregulated in the Δ*cpc1* mutant strain (Fig. 1E).

Detached leaves of susceptible *Zea mays* cultivar B73 were inoculated with mycelial plugs of WT strain C4, Δ*cpc1* mutants, and complemented strains. Compared with WT and the complemented strains, Δ*cpc1* mutants caused smaller lesions on 2 and 3 dpi (Fig. 1F and G). Thus, *CPC1* was necessary for the virulence of *C. heterostrophus*.

### ChCpc1 was necessary for nitrogen assimilation and response to amino acid starvation during conidiation and mycelial growth

**Fig 2:**
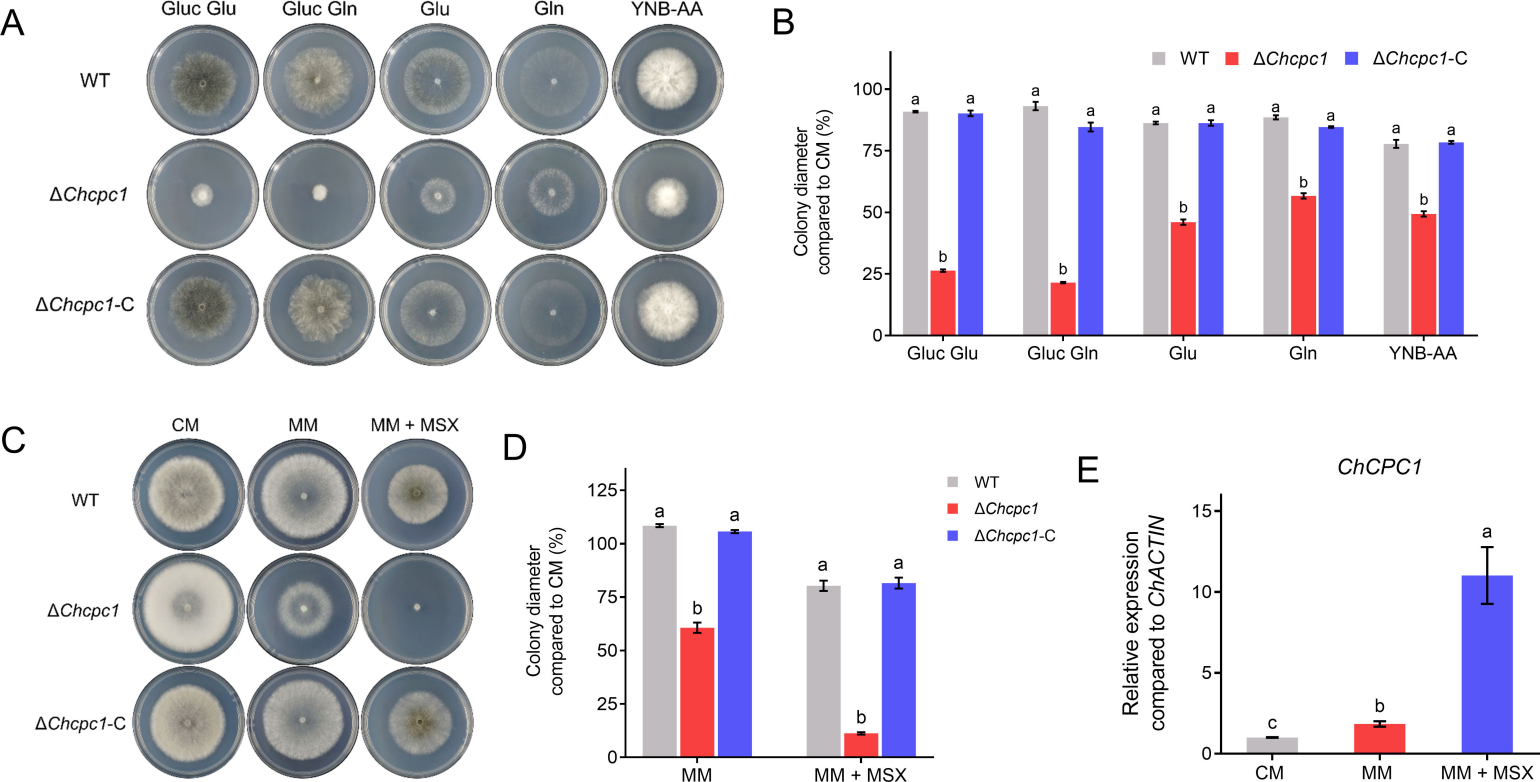
ChCpc1 was necessary for nitrogen assimilation and response to amino acid starvation during mycelial growth. (**A**) Plate tests showing mycelial growth of WT, Δ*cpc1* mutants, and complemented strains on minimal media supplemented with the indicated carbon or nitrogen sources and YNB-AA. (**B**) Statistical analysis of mycelial growth of WT, Δ*cpc1* mutants, and complemented strains on minimal media supplemented with the indicated carbon or nitrogen sources and YNB-AA. (**C**) Radial growth of WT, Δ*cpc1* mutants, and complemented strains on complete medium (CM), minimal medium (MM), and MM supplemented with 100 µM L-methionine-DL-sulfoximine (MSX). (**D**) Statistical analysis of radial growth of WT, Δ*cpc1* mutants, and complemented strains on CM, MM, and MM supplemented with MSX. (**E**) Relative expression of *ChCPC1* on different media. Gluc Glu: glucose-containing media supplemented with glutamate as the sole nitrogen source; Gluc Gln: glucose-containing media supplemented with glutamine as the sole nitrogen source; Glu: glutamate as carbon sources; Gln: glutamine as carbon sources; YNB-AA: yeast nitrogen base without amino acid. GraphPad Prism program’s *t-*tests and multiple *t-*tests were used for the analysis of significant differences. The bars indicate the standard error of the means of three replications.

To determine the role of ChCpc1 in nitrogen assimilation, the WT, the Δ*cpc1* mutants, and the complemented strains were inoculated on glucose-containing media supplemented with glutamine or glutamate as the sole nitrogen source (10). As shown in Fig. 2, the radial growth of Δ*cpc1* mutants was almost abolished on glucose-containing media supplemented with glutamine or glutamate as the sole nitrogen source (Fig. 2A and B). Thus, the deletion of *ChCPC1* caused the inability of *C. heterostrophus* to use glutamine or glutamate as nitrogen sources for growth and N assimilation. In addition, to determine the role of ChCpc1 in glutaminolysis, the WT, the Δ*cpc1* mutants, and the complemented strains were inoculated on MM supplemented with glutamine or glutamate. The radial growth of Δ*cpc1* mutants was impaired on MM that contained glutamine or glutamate (Fig. 2A and B). This proved that Δ*cpc1* mutants were impaired in both glutaminolysis and synthesis of nitrogen-containing compounds. Moreover, to further confirm the function of ChCpc1 in amino acid synthesis and response to amino acid starvation, the WT, the Δ*cpc1* mutants, and the complemented strains were inoculated on yeast nitrogen base without amino acid (YNB-AA). The reduced mycelial growth of the Δ*cpc1* mutants suggested that ChCpc1 was involved in amino acid synthesis and response to amino acid starvation (Fig. 2A and B).

To confirm the role of ChCpc1 in amino acid synthesis and response to amino acid starvation, the WT, the Δ*cpc1* mutants, and the complemented strains were inoculated on MM or MM supplemented with 100 µM MSX (L-methionine-DL-sulfoximine, which is an amino acid synthesis inhibitor). As evidenced by the abolished colony radial growth of Δ*cpc1* mutants and the upregulated expression levels of *ChCPC1* on MM supplemented with MSX, we confirmed that ChCpc1 was involved in amino acid synthesis and response to amino acid starvation in *C. heterostrophus* (Fig. 2C through E).

### Metabolomics analysis of the Δ*cpc1* mutant after amino acid deprivation

**Fig 3:**
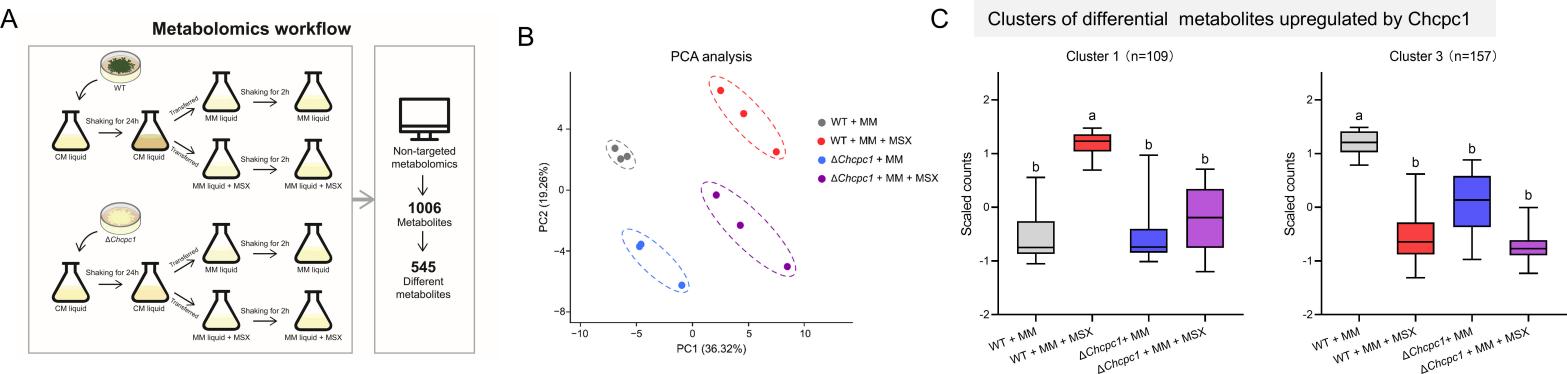
Metabolomics analysis of the Δ*cpc1* mutant after amino acid deprivation. (**A**) Metabolomics workflow. (**B**) Principal component analysis of the metabolomics data sets. (**C**) Identified clusters of differential metabolites upregulated by ChCpc1 through K-means clustering analysis. GraphPad Prism program’s *t-*tests and multiple *t-*tests were used for the analysis of significant differences.

To investigate the effect of ChCpc1 on metabolites in *C. heterostrophus*, metabolomics analysis was performed. Four types of samples were included in the metabolomics analysis: WT cultured on MM, WT cultured on MM with MSX, Δ*cpc1* mutant cultured on MM, and Δ*cpc1* mutant cultured on MM with MSX (Fig. 3A). Principal component analysis (PCA) proved that there was good reproducibility among biological replicates for each sample (Fig. 3B). Volcano diagrams were used to explore the differentially accumulated metabolites (DAMs), and four comparisons were conducted, namely, WT_MM_MSX with WT_MM, Δ*cpc1*_MM_MSX with Δ*cpc1*_MM, Δ*cpc1*_MM_MSX with WT_MM_MSX, and Δ*cpc1*_MM with WT_MM. Compared with WT_MM, a total of 209 DAMs were identified, including 105 upregulated metabolites and 104 downregulated metabolites in WT_MM_MSX. Under the same MM culture conditions, 91 and 60 metabolites with increased and decreased abundance, respectively, were identified in the Δ*cpc1* mutant compared to WT. MSX exposure caused 71 and 125 metabolites to be up- and downregulated in the Δ*cpc1* mutant, respectively. However, in the presence of MSX, more metabolites increased and accumulated in the Δ*cpc1* mutant compared with WT (Table S1).

**Fig 4:**
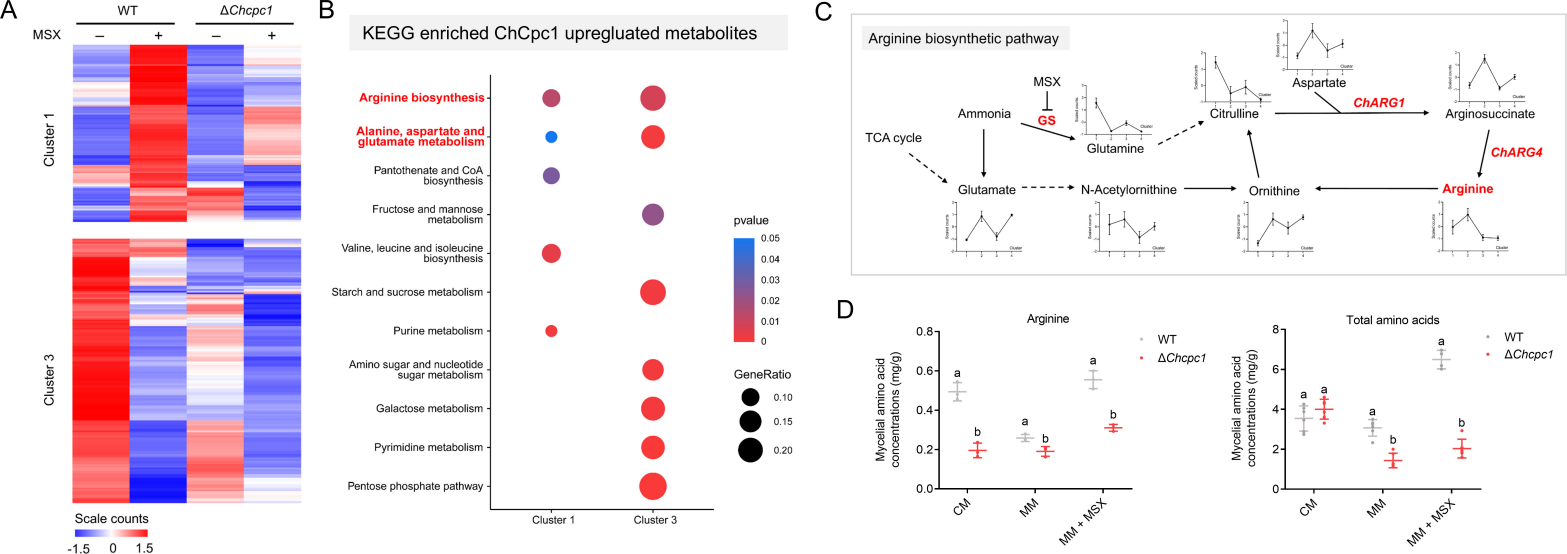
Clustering and KEGG analysis of the differential metabolites in WT and the Δ*cpc1* mutant. (**A**) Clustering heatmap and (**B**) KEGG functional enrichment analysis of the differential metabolites in WT and the Δ*cpc1* mutant in the presence of L-methionine-DL-sulfoximine (MSX) or not. (**C**) Proposed arginine synthesis pathway in *Cochliobolus heterostrophus*. (**D**) Loss of *ChCPC1* resulted in a notable reduction in arginine and total amino acid in liquid MM, whether supplemented with MSX or not. CM: complete medium; MSX: L-methionine-DL-sulfoximine; MM: minimal medium; MM + MSX: minimal medium supplemented with 100 µM L-methionine-DL-sulfoximine (MSX). GraphPad Prism program’s *t-*tests and multiple *t-*tests were used for the analysis of significant differences.

All the relative contents of DAMs were standardized by a *z*-score, and then K-means cluster analysis was performed; four clusters were identified, and the trends in the variation of DAMs were different in each cluster (Fig. S2). For example, Cluster 1 contained 109 DAMs that increased only in WT treated with MSX, and Cluster 3 contained 157 DAMs that exhibited the highest content levels in WT cultured in MM (Fig. 3C and 4A). However, all these DAMs in clusters 1 and 3 showed no significant changes in the Δ*cpc1* mutant. This result demonstrated that these DAMs were underregulated by ChCpc1.

To determine the functions of these Cpc1-regulated DAMs further, we conducted the KEGG analysis. In the presence of MSX, several pathways were regulated by ChCpc1, which included amino acid synthesis, pantothenate and CoA biosynthesis, and purine metabolism (Fig. 4B). In addition, ChCpc1 also influenced fructose and mannose metabolism, galactose metabolism, and pyrimidine metabolism pathways. Most notably, both the arginine synthesis and alanine, aspartate, and glutamate metabolism (marked in red) pathways were significantly enriched in clusters 1 and 3, reflecting the critical role of arginine and other amino acids in the nitrogen assimilation process of fungal pathogens (Fig. 4B).

Finally, we predicted the arginine synthesis pathway in *C. heterostrophus* by referring to the pathway in *Cochliobolus carbonum*. In the presence of MSX, the activity of glutamine synthetase was inhibited, which led to a significant reduction in glutamine content, and the content of its downstream product citrulline also decreased. In contrast, MSX treatment significantly increased the concentration of glutamate, N-acetylornithine, and ornithine in another nitrogen assimilation pathway. Moreover, compared with WT, the concentration of intermediate product arginosuccinate and end-product arginine in the Δ*cpc1* mutant was remarkably reduced when exposed to MSX (Fig. 4C). Considering the direct roles of *ChARG1* and *ChARG4* in arginine synthesis, we speculated that ChCpc1 regulated the expression of these two genes positively.

To verify the results of the metabolomics analysis, the amino acid concentration of WT and the Δ*cpc1* mutant was determined using the arginine (Arg) and amino acid (AA) content assay kits. Consistent with the metabolomics results, the loss of *ChCPC1* resulted in a notable reduction in arginine and total amino acids in liquid MM, regardless of whether it was supplemented with MSX or not (Fig. 4D). These results revealed that ChCpc1 increased the amino acid concentration in the nitrogen assimilation process to cope with nutrient deficiency in *C. heterostrophus*.

### ChCpc1 activated the arginine synthesis to regulate conidiation and virulence in *C. heterostrophus*

**Fig 5:**
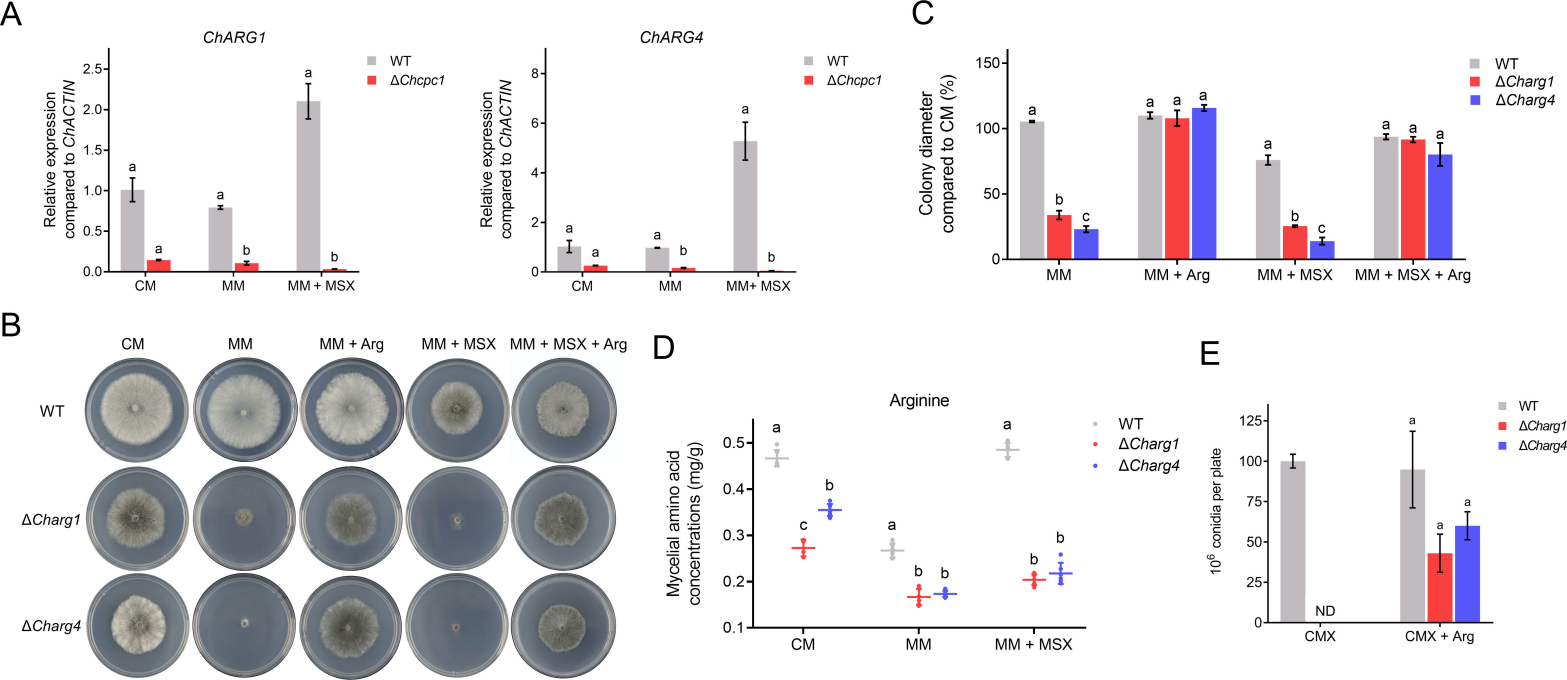
ChArg1 and ChArg4 involved in response to amino acid starvation. (**A**) Expression levels of *ChARG1* and *ChARG4* in WT and Δ*cpc1* mutants under different culture conditions. (**B**) Radial growth of WT, Δ*Charg1*, and Δ*Charg4* mutants on indicated medium. (**C**) Statistical analysis of radial growth of WT, Δ*Charg1*, and Δ*Charg4* mutants on indicated medium. (**D**) Arginine concentration of WT, Δ*Charg1*, and Δ*Charg4* mutants cultured on indicated medium. (**E**) Conidiation of WT, Δ*Charg1*, and Δ*Charg4* mutants on CMX and CMX supplemented with exogenous arginine. CM: complete medium; MM: minimal medium; MM + MSX: minimal medium supplemented with 100 µM L-methionine-DL-sulfoximine (MSX); MM + Arg: minimal medium supplemented with 10 mM arginine; MM + MSX + arg: minimal medium supplemented with 100 µM MSX and 10 mM arginine; CMX: complete medium with xylose; CMX + Arg: CMX supplemented with arginine. GraphPad Prism program’s *t-*tests and multiple *t-*tests were used for the analysis of significant differences. The bars indicate the standard error of means of three replications.

To explore the regulatory mechanism of ChCpc1 on arginine synthesis, the expression levels of arginine synthesis-related genes were detected in WT and Δ*cpc1* mutants. The results showed that MSX significantly induced the expressions of *ChARG1* and *ChARG4* in WT. However, the expressions of *ChARG1* and *ChARG4* were completely inhibited in Δ*cpc1* mutants, even in the presence of MSX (Fig. 5A). This indicated that ChCpc1 regulated arginine synthesis directly to increase its capability to address amino acid starvation.

In addition, the Δ*arg1* and Δ*arg4* mutants exhibited similar phenotypes on MM or MM supplemented with 100 µM MSX. Δ*arg1* and Δ*arg4* mutants were unable to grow on MM and MM with MSX, but exogenous arginine (10 mM) restored the growth of hyphae in the Δ*arg1* and Δ*arg4* mutants on MM and MM with MSX (Fig. 5B and C). Furthermore, the concentrations of arginine in Δ*arg1* and Δ*arg4* mutants were significantly lower than those in the WT under different culture media (Fig. 5D).

**Fig 6:**
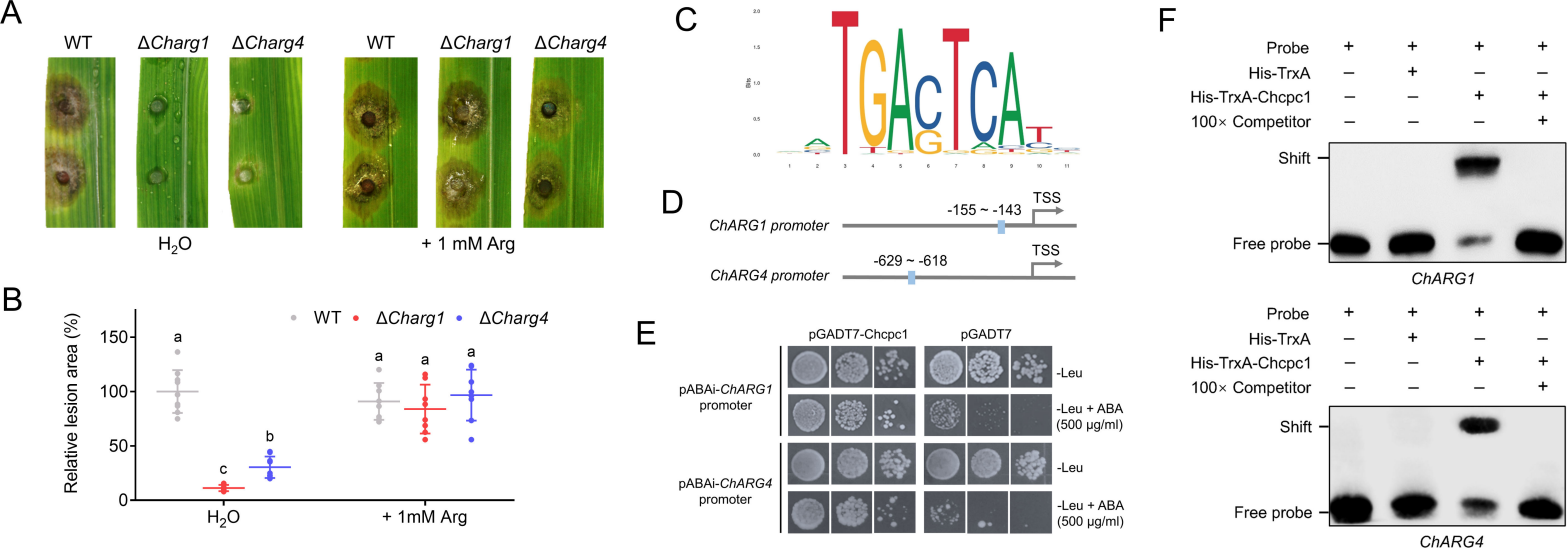
ChCpc1 activated arginine synthesis to regulate virulence in *Cochliobolus heterostrophus*. (**A**) Leaves showing disease symptoms caused by WT, Δ*Charg1*, and Δ*Charg4* mutants in the presence or absence of exogenous arginine. (**B**) Statistical analysis of the lesion size caused by WT, Δ*Charg1*, and Δ*Charg4* mutants in the presence or absence of exogenous arginine. (**C**) The putative sequence of the *cis*-element in the promoters of ChCpc1 target genes. (**D**) Schematic representation of the *cis*-element in the promoters of *ChARG1* and *ChARG4*. (**E**) Binding of ChCpc1 to the promoter of *ChARG1/4* in yeast one-hybrid assay. (**F**) Verification of the binding of ChCpc1 with the *cis*-element by electrophoretic mobility shift assay (EMSA) and competitive probes (100×) that were used for competition experiments. GraphPad Prism program’s *t-*tests and multiple *t-*tests were used for the analysis of significant differences. The bars indicate the standard error of means of three replications.

To evaluate whether the arginine auxotrophic phenotypes in conidiation could be rescued by the addition of exogenous arginine, Δ*arg1* and Δ*arg4* mutants were grown on CMX or CMX supplemented with arginine. The addition of exogenous arginine in CMX partially restored the conidiation of Δ*arg1* and Δ*arg4* mutants (Fig. 5E). Moreover, 1 mM of arginine could completely rescue the virulence of Δ*arg1* and Δ*arg4* mutants on detached maize leaves (Fig. 6A and B). These results demonstrated that *ChARG1* and *ChARG4* played important roles in conidiation and virulence in *C. heterostrophus*.

To verify whether ChCpc1 binds to the promoter region of *ChARG1* and *ChARG4*, we conducted *cis*-acting element prediction and Y1H. The results demonstrated that ChCpc1 bound to the predicted promoter region in the *ChARG1/4* promoter (Fig. 6C and D). To further confirm the binding of ChCpc1 to the promoter region of *ChARG1/4*, we conducted the electrophoretic mobility shift assay (EMSA). The EMSA demonstrated that ChCpc1-His bound to the *ChARG1/4*^pro^, with excess unlabeled competitors preventing this binding (Fig. 6F). Additionally, Y1H further confirmed ChCpc1 bound to the promoter regions of *ChARG1* and *ChARG4* (Fig. 6E). All these results confirmed the specificity of the interaction between ChCpc1 and *ChARG1/4*.

### ChCpc1 required for amino acid starvation induced autophagy in *C. heterostrophus*

**Fig 7:**
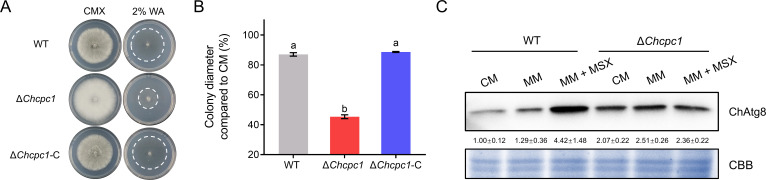
ChCpc1 was required for autophagy induction in *C. heterostrophus* under amino acid deprivation. (**A**) Radial growth of WT, Δ*cpc1* mutants, and complemented strain on CMX and 2% WA. (**B**) Statistical analysis of radial growth of WT, Δ*cpc1* mutants, and complemented strain on CMX and 2% WA. (**C**) Accumulation of ChAtg8 in WT and Δ*cpc1* mutants under different culture conditions. 2% WA: 2% water agar; CM: complete medium; MSX: L-methionine-DL-sulfoximine; MM: minimal medium; MM + MSX: minimal medium supplemented with 100 µM L-methionine-DL-sulfoximine (MSX); CMX: complete medium with xylose; GraphPad Prism program’s *t*-tests and multiple *t-*tests were used for the analysis of significant differences. The bars indicate the standard error of means of three replications.

Generally, autophagy is induced by amino acid starvation. To investigate the regulatory role of ChCpc1 in autophagy, WT and Δ*cpc1* mutants were inoculated on 2% water agar (WA). Compared with WT, the Δ*cpc1* mutants exhibited slower radial growth on 2% WA (Fig. 7A and B). This indicated that autophagy may be suppressed in the absence of ChCpc1 in *C. heterostrophus*. To better understand the role of ChCpc1 in autophagy induction under amino acid starvation, GFP-ChAtg8 was transformed into WT and Δ*cpc1* mutants. Western blot analysis with anti-Atg8 antibody showed that MSX treatment significantly increased the accumulation of Atg8 in WT. However, 100 µM MSX did not induce accumulation of Atg8 in Δ*cpc1* mutants (Fig. 7C). These results indicated that amino acid starvation induced autophagy in *C. heterostrophus*, which depends on the presence of ChCpc1.

**Fig 8:**
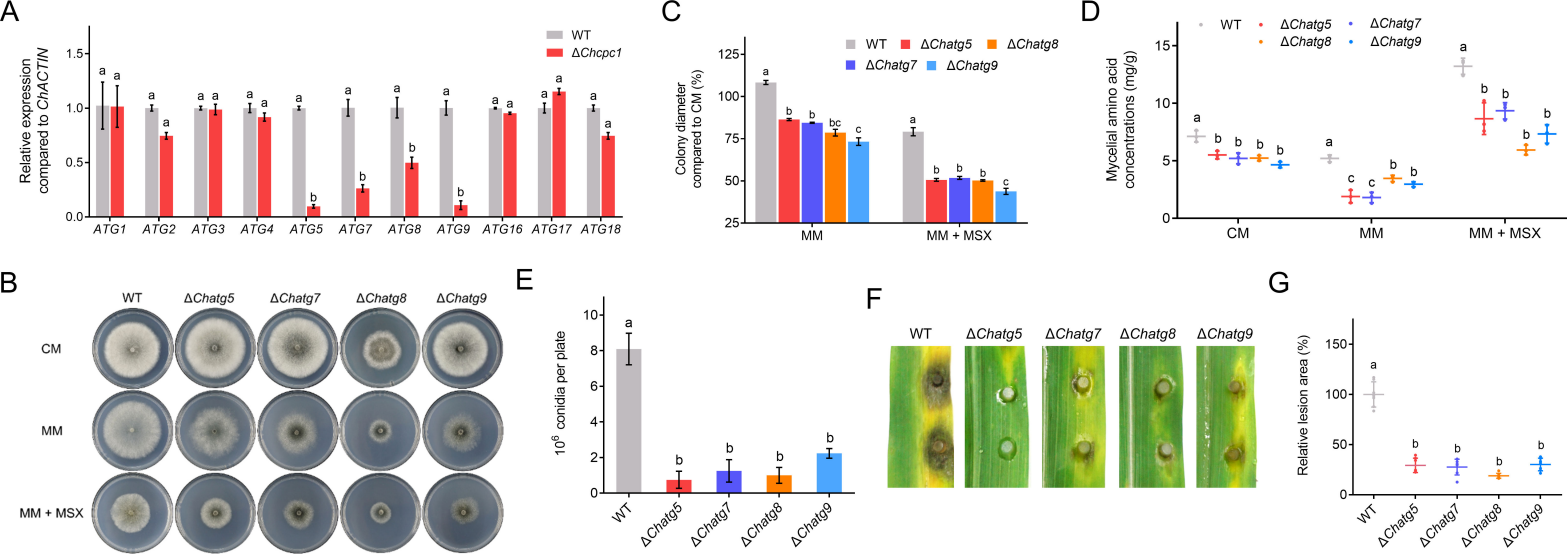
*ChATG5*, *ChATG7*, *ChATG8*, and *ChATG9* are necessary for conidiation, virulence, and response to amino acid starvation in *Cochliobolus heterostrophus*. (**A**) Expression levels of *ChATGs* in WT and Δ*cpc1* mutants under amino acid starvation conditions. (**B**) Radial growth of WT, Δ*atg5*, Δ*atg7*, Δ*atg8*, and Δ*atg9* on complete medium (CM), minimal medium (MM), and MM supplemented with L-methionine-DL-sulfoximine (MSX). (**C**) Statistical analysis of radial growth of WT, Δ*atg5*, Δ*atg7*, Δ*atg8*, and Δ*atg9* on complete medium (CM), minimal medium (MM), and MM supplemented with 100 µM MSX. (**D**) Amino acid concentration of WT, Δ*atg5*, Δ*atg7*, Δ*atg8*, and Δ*atg9* cultured on different media. (**E**) Statistical analysis of conidiation of WT, Δ*atg5*, Δ*atg7*, Δ*atg8*, and Δ*atg9*. (**F**) Leaves showing disease symptoms caused by WT, Δ*atg5*, Δ*atg7*, Δ*atg8*, and Δ*atg9*. (**G**) Statistical analysis of the lesion size caused by WT, Δ*atg5*, Δ*atg7*, Δ*atg8*, and Δ*atg9*. MM: minimal medium; MM + MSX: minimal medium supplemented with 100 µM L-methionine-DL-sulfoximine (MSX); GraphPad Prism program’s *t-*tests and multiple *t-*tests were used for the analysis of significant differences. The bars indicate the standard error of means of three replications.

To further understand how ChCpc1 was involved in autophagy under amino acid deprivation, we first assessed the expression levels of 11 *ChATG* genes in WT and Δ*cpc1* mutants. Four genes (*ChATG5*, *ChATG7*, *ChATG8*, and *ChATG9*) showed decreased expression levels in Δ*cpc1* mutants compared with WT in the presence of 100 µM MSX (Fig. 8A). Then, *ChATG5*, *ChATG7*, *ChATG8*, and *ChATG9* were knocked out in WT C4. We compared the colony diameters of Δ*Chatg5*, Δ*Chatg7*, Δ*Chatg8*, and Δ*Chatg9* mutants with WT on CM, MM, and MM with 100 µM MSX. When grown on CM, there were almost no significant differences in radial growth between the WT and Δ*atg* mutants, except for Δ*Chatg8* (Fig. 8B).

However, when cultured on MM or MM supplemented with 100 µM MSX, mycelial growth was significantly impaired in Δ*atg* mutants (Fig. 8B and C). The amino acid concentration of Δ*Chatg5*, Δ*Chatg7*, Δ*Chatg8*, and Δ*Chatg9* mutants was significantly lower than that in WT under different culture conditions (Fig. 8D). These data indicated that *ChATG5*, *ChATG7*, *ChATG8*, and *ChATG9* were involved in the response to amino acid starvation.

**Fig 9:**
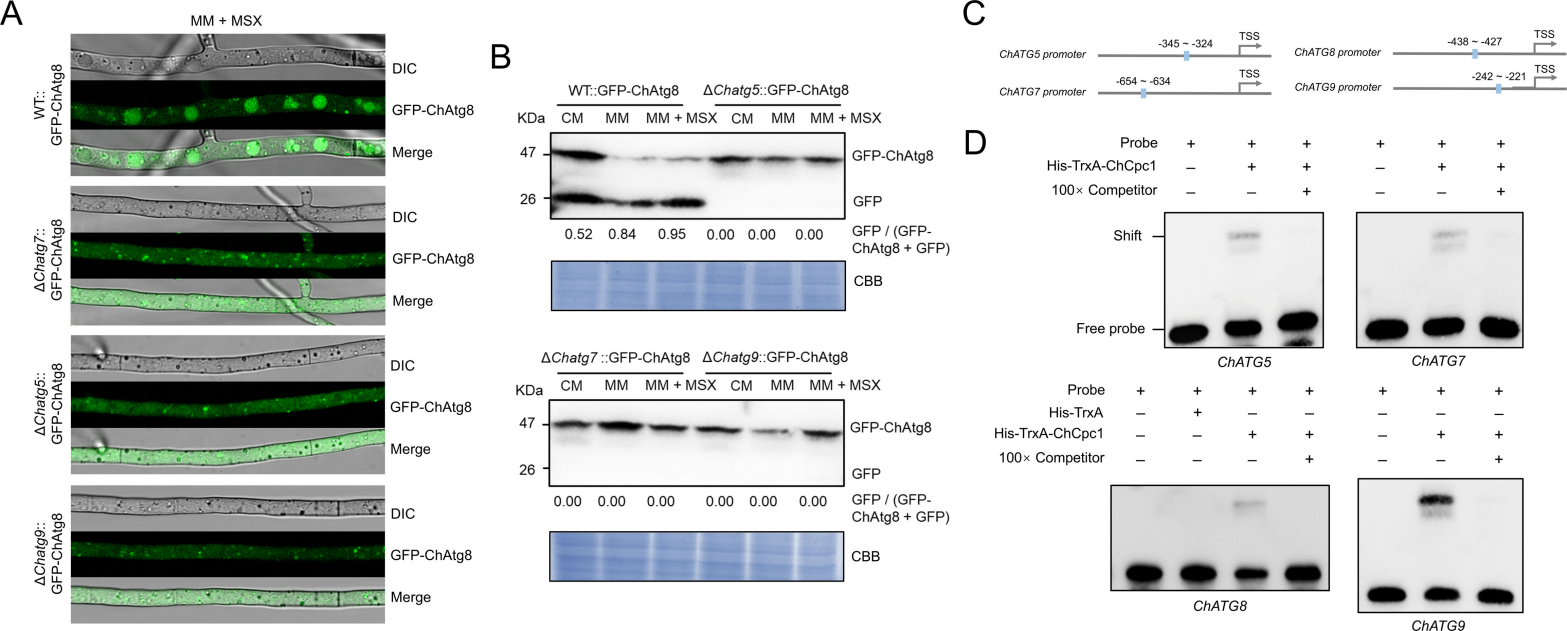
ChCpc1 bound to the promoter regions of *ChATG* genes to regulate autophagy in *Cochliobolus heterostrophus*. (**A**) Detection of GFP-Atg8 in WT, Δ*atg5*, Δ*atg7*, and Δ*atg9* cultured in MM supplemented with 100 µM MSX. (**B**) GFP-Atg8 proteolysis analysis of WT, Δ*atg5*, Δ*atg7*, and Δ*atg9* cultured in CMX, MM, and MM supplemented with 100 µM MSX. (**C**) Schematic representation of the *cis*-element in the promoters of *ChATG5*, *ChATG7*, *ChATG8*, and *ChATG9*. (**D**) Verification of the binding of ChCpc1 with the *cis*-element by electrophoretic mobility shift assay (EMSA) and competitive probes (100×) were used for competition experiments. MM + MSX: minimal medium supplemented with 100 µM L-methionine-DL-sulfoximine (MSX); GraphPad Prism program’s *t*-tests and multiple *t-*tests were used for the analysis of significant differences. The bars indicate the standard error of means of three replications.

Furthermore, Δ*Chatg5*, Δ*Chatg7*, Δ*Chatg8*, and Δ*Chatg9* mutants showed a dramatic decrease in conidiation at 7 days compared with WT (Fig. 8E). Inoculation with mycelial plugs from Δ*Chatg5*, Δ*Chatg7*, Δ*Chatg8*, and Δ*Chatg9* mutants resulted in smaller lesions on detached leaves compared with WT, which demonstrated that *ChATG5*, *ChATG7*, *ChATG8*, and *ChATG9* were necessary for the virulence of *C. heterostrophus* (Fig. 8F and G). In addition, the accumulation of GFP-ChAtg8 in vacuoles was obviously blocked in Δ*atg5*, Δ*atg7*, and Δ*atg9* mutants under amino acid deprivation (Fig. 9A). Moreover, the cleavage of GFP-ChAtg8 was nearly abolished in Δ*atg5*, Δ*atg7*, and Δ*atg9* mutants under amino acid deprivation (Fig. 9B).

To explore the regulatory mechanism of ChCpc1 on autophagy, we validated the relationship between ChCpc1 and *ChATG* genes using EMSA. The results showed that ChCpc1 bound to the putative promoter region of all four genes, and ChCpc1 exhibited the strongest binding with the promoter of *ChATG9* (Fig. 9C and D). These results indicated that ChCpc1 was capable of binding to the promoter region of *ChATG* under amino acid starvation in *C. heterostrophus*.

### ChAtf1 enhanced the binding ability of ChCpc1 to the promoter regions of *ChARG4* and *ChATG8*

**Fig 10:**
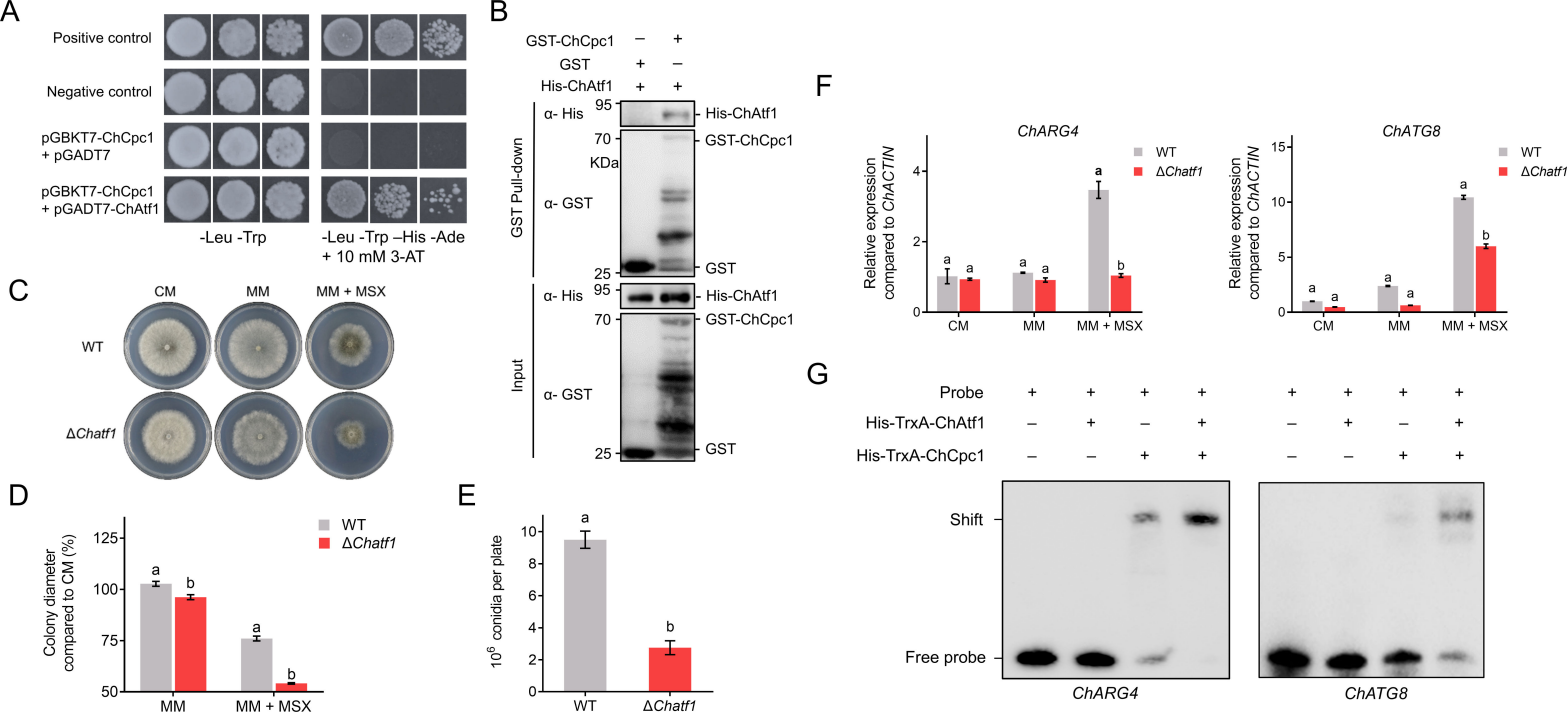
ChAtf1 enhanced the binding ability of ChCpc1 to the promoter of *ChARG4* and *ChATG8*. (**A**) ChCpc1 interacted with ChAtf1 in a yeast two-hybrid assay. Serial dilutions of yeast cells were plated on synthetic dropout (SD) medium that lacked adenine (A), histidine (H), leucine (L), and tryptophan (T) (SD-A/H/L/T). The interaction between pGADT7-T and pGBKT7-53 was used as positive control, while that between pGADT7-T and pGBKT7-Lam was used as negative control. (**B**) Pull-down assay to verify the interaction between ChCpc1 and ChAtf1. The fusion protein His-Atf1 was detected in the eluted solution of GST-ChCpc1 and His-ChAtf1 co-incubated mixture but not in that of glutathione S-transferase (GST) or His-ChAtf1. (**C**) Radial growth of WT and Δ*atf1* on CM, MM, and MM supplemented with 100 µM MSX. (**D**) Statistical analysis of radial growth of WT and Δ*atf1* on MM and MM supplemented with MSX compared with CM. (**E**) Conidiation of WT and Δ*atf1* mutants. (**F**) Expression levels of *ChARG4* and *ChATG8* in WT and Δ*atf1* mutants under different culture conditions. (**G**) Electrophoretic mobility shift assay confirmed the enhanced binding effect of ChCpc1 to the promoter region of *ChARG4* and *ChATG8* in the presence of ChAtf1. CM: complete medium; MSX: L-methionine-DL-sulfoximine; MM: minimal medium; MM + MSX: minimal medium supplemented with 100 µM L-methionine-DL-sulfoximine (MSX). GraphPad Prism program’s *t*-tests and multiple *t-*tests were used for the analysis of significant differences. The bars indicate the standard error of means of three replications.

Through yeast two-hybrid (Y2H) library screening, we identified another bZIP transcriptional factor, ChAtf1. To verify the interaction between ChCpc1 and ChAtf1, Y2H and GST pull-down assays were further conducted. As shown in Fig. 10A, yeast strains co-transformed with pGBKT7-ChCpc1 and pGADT7-ChAtf1 grew well on the SD-A/H/L/T medium. In addition, the interaction between ChCpc1 and ChAtf1 was confirmed by a GST pull-down assay (Fig. 10B).

To further investigate the role of ChAtf1 in conidiation upon amino acid starvation, the Δ*Chatf1* mutant strains were constructed. Similar to the Δ*Chcpc1* mutant strains, the Δ*Chatf1* mutant strains exhibited a reduced mycelial growth rate on MM supplemented with 100 µM MSX (Fig. 10C and D). In addition, compared with WT, the Δ*Chatf1* mutants showed a dramatic decrease in conidiation (Fig. 10E). Moreover, the expression levels of *ChARG4* and *ChATG8* in Δ*Chatf1* were significantly inhibited by MSX (Fig. 10F). Unlike Δ*Chcpc1*, the deletion of *ChATF1* had no effect on the expression of *ChATG5*, *ChATG7*, or *ChATG9*. However, ChATF1 had no direct regulatory function on *ChARG4* or *ChATG8* (data not shown). These results suggested that ChAtf1 also responded to amino acid starvation, which implied that ChAtf1 was involved in the regulation of nitrogen utilization in *C. heterostrophus*.

Even though ChAtf1 did not bind to the promoter regions of *ChARG4* or *ChATG8*, we next asked whether ChAtf1 enhanced the binding effect of ChCpc1. Considering that ChCpc1 may form a heterodimer with ChAtf1 to regulate the target genes, purified proteins of ChCpc1 and ChAtf1 were mixed and incubated with the promoter regions of *ChARG4* and *ChATG8* to determine the binding effect using EMSA. Finally, the presence of ChAtf1 enhanced the binding effect of ChCpc1 on the promoters of *ChARG4* and *ChATG8* dramatically (Fig. 10G). That is, although ChAtf1 did not bind directly to the promoter regions of *ChARG4* or *ChATG8*, it enhanced the binding effect of ChCpc1 to the *ChARG4* and *ChATG8* promoter by interacting with ChCpc1 to increase transcription of *ChARG4* and *ChATG8*.

### Mitogen-activated protein kinase ChChk1 phosphorylated ChCpc1 to counteract amino acid depletion

**Fig 11:**
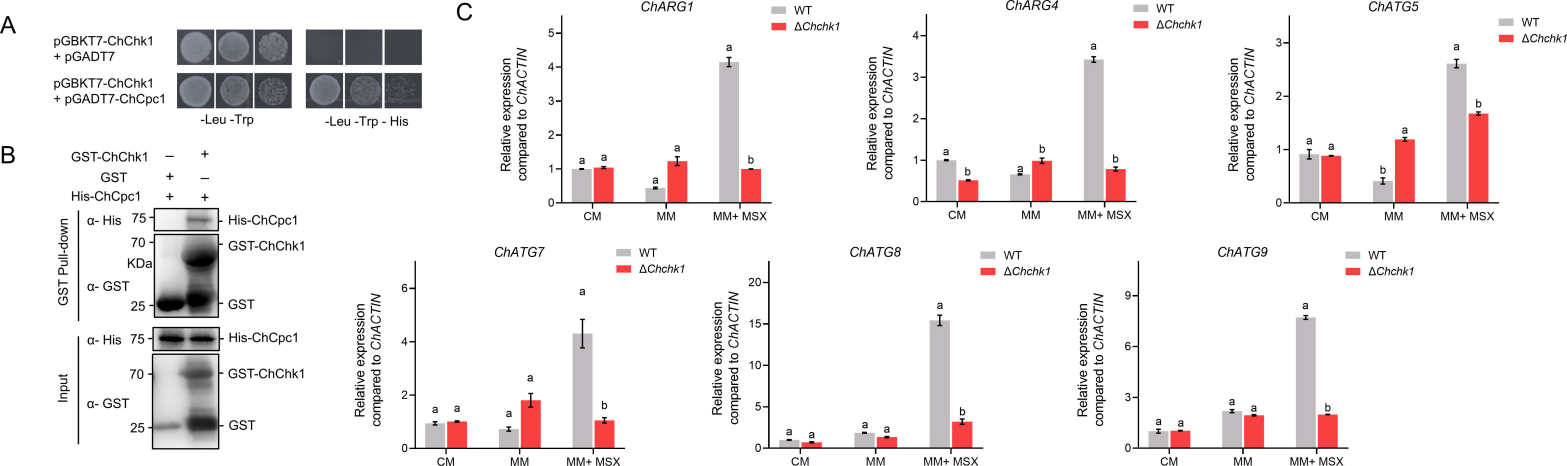
ChCpc1 interacted with ChChk1 in *Cochliobolus heterostrophus*. (**A**) ChCpc1 interacted with ChChk1 in yeast two-hybrid assay. Serial dilutions of the yeast cells were plated on synthetic dropout (SD) medium that lacked histidine (H), leucine (L), and tryptophan (**T**) (SD- T/H/L). The interaction between pGADT7-T and ChChk1 was used as control. (**B**) Pull-down assay to verify the interaction between ChCpc1 and ChChk1. The fusion protein His-Cpc1 was detected in the eluted solution of GST-ChChk1 and His-ChCpc1 co-incubated mixture but not in that of glutathione S-transferase (GST) and His-ChCpc1. (**C**) Expression levels of *ChARG1*, *ChARG4*, *ChATG5*, *ChATG7*, *ChATG8*, and *ChATG9* in WT and Δ*chk1* mutants under different culture conditions. CM: complete medium; MSX: L-methionine-DL-sulfoximine; MM: minimal medium; MM + MSX: minimal medium supplemented with 100 µM L-methionine-DL-sulfoximine (MSX). GraphPad Prism program’s *t-*tests and multiple *t-*tests were used for the analysis of significant differences. The bars indicate the standard error of means of three replications.

The mitogen-activated protein kinase ChChk1 was identified through Y2H library screening using ChCpc1 as bait. The results demonstrated the interaction between ChChk1 and ChCpc1 in yeast, as detailed in Fig. 11A. In addition, glutathione S-transferase (GST)-tagged ChChk1 and His-tagged ChCpc1 were transformed into *Escherichia coli* BL21(DE3), and the GST pull-down assays confirmed that GST-ChChk1 interacted with His-ChCpc1 (Fig. 11B).

**Fig 12:**
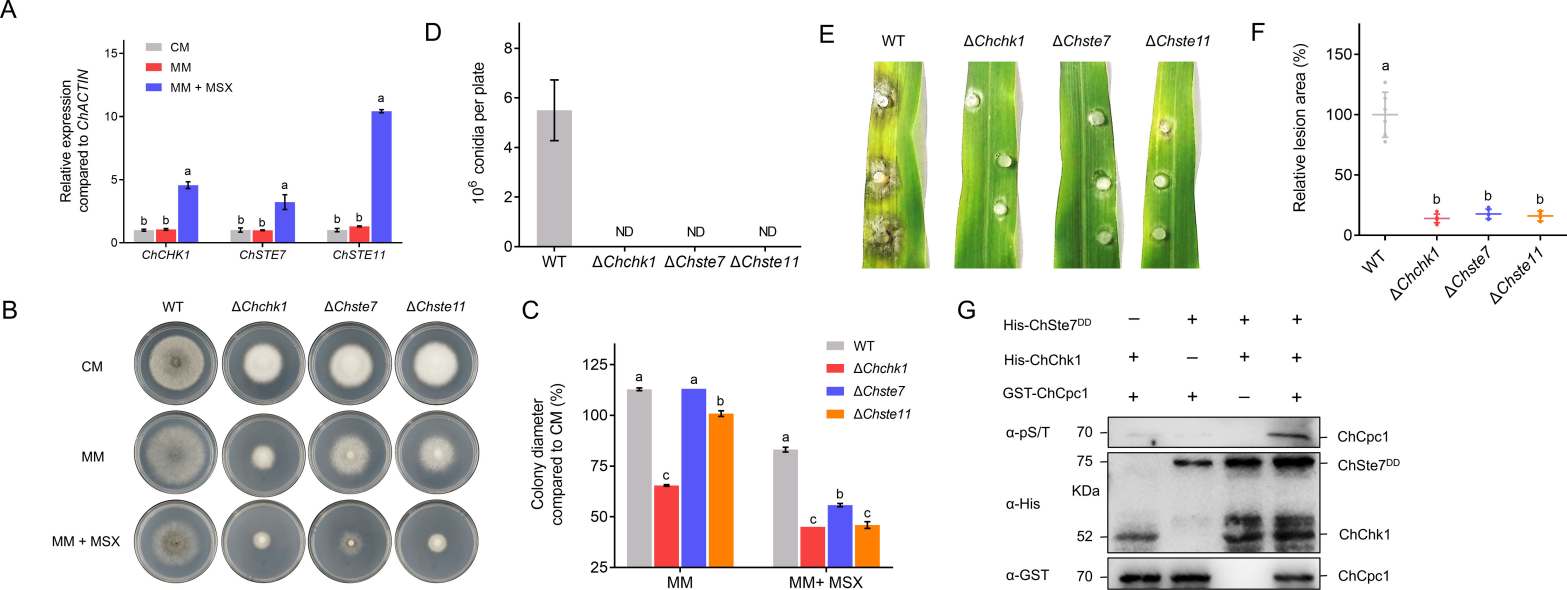
ChChk1 phosphorylates ChCpc1 in *Cochliobolus heterostrophus*. (**A**) Expression levels of *ChCHK1*, *ChSTE7*, and *ChSTE11* were induced by MSX. (**B**) Mycelial growth of Δ*Chchk1*, Δ*Chste11*, and Δ*Chste7* was inhibited significantly by MSX compared with WT. (**C**) Statistical analysis of the mycelial growth of Δ*Chchk1*, Δ*Chste11*, and Δ*Chste7* on MM or MM amended with MSX. (**D**) Conidiation of WT and Δ*Chchk1*, Δ*Chste11*, and Δ*Chste7* mutants. (**E**) Detached maize leaves that showed disease symptoms caused by WT, Δ*Chchk1*, Δ*Chste11*, and Δ*Chste7*. (**F**) Statistical analysis of the lesion size caused by WT, Δ*Chchk1*, Δ*Chste11*, and Δ*Chste7*. (**G**) Western blot analysis of an *in vitro* phosphorylation experiment between ChChk1 and ChCpc1. Proteins were immunoblotted with appropriate antisera (listed on the right). CM: complete medium; MSX: L-methionine-DL-sulfoximine; MM: minimal medium; MM + MSX: minimal medium supplemented with 100 µM L-methionine-DL-sulfoximine (MSX). GraphPad Prism program’s *t-*tests and multiple *t-*tests were used for the analysis of significant differences. The bars indicate the standard error of means of three replications.

Similar to Δ*Chcpc1* mutant strains, the deletion of *ChCHK1* also inhibited the expressions of *ChARG1*, *ChARG4*, *ChATG5*, *ChATG7*, *ChATG8*, and *ChATG9* under amino acid depletion (Fig. 11C). Considering that *ChCHK1*, *ChSTE7*, and *ChSTE11* are important components of the MAPK pathway, we examined the expressions of *ChCHK1*, *ChSTE7*, and *ChSTE11*. The results showed that MSX significantly induced the expressions of *ChCHK1*, *ChSTE7*, and *ChSTE11* (Fig. 12A). In addition, the mycelial growth of the mutants was also determined on CM supplemented with MSX. The results showed that the mycelial growth of Δ*Chchk1*, Δ*Chste11*, and Δ*Chste7* was dramatically inhibited by MSX compared to WT (Fig. 12B and C). Moreover, the conidiation and virulence of Δ*Chchk1*, Δ*Chste11*, and Δ*Chste7* were completely abolished (Fig. 12D through F).

Considering that ChChk1 is a protein kinase and interacts with ChCpc1, we investigated whether ChCpc1 was a direct phosphorylated target of ChChk1. An *in vitro* phosphorylation assay was used to determine the association between ChChk1 and ChCpc1, and ChSte7^DD^ (Ser-216 and Thr-220 were replaced by Asp) was used to activate ChChk1. In the phosphorylation assay, the phosphorylation level of ChCpc1 increased significantly in the presence of ChSte7^DD^ (Fig. 12G), which proved that ChChk1 phosphorylated ChCpc1.

### Identifying *ChCPC1*- and *ChATF1*-dependent changes in gene expression of *C. heterostrophus*

To delve into the roles of *ChCPC1* and *ChATF1* in *C. heterostrophus*, we compared the transcriptomes of Δ*Chcpc1* or Δ*Chatf1* with that of WT by RNA-seq using three biological replicates. Feature Counts v. 1.5.0-p3 was used to count the numbers of reads that were mapped to each gene. Then, the fragments per kilobase million (FPKM) of each gene were calculated based on the length of the gene and the number of reads mapped to the respective gene. PCA was conducted with FPKM to test the reproducibility between the biological replicates of each sample (Fig. S3A). In addition, Pearson’s correlation coefficient test showed that there was a good correlation among the biological replicates (Fig. S3B).

In Δ*Chcpc1* compared with WT, 1,541 differentially expressed genes (DEGs) were identified in three replicates, which included 614 upregulated and 927 downregulated genes (Fig. S4A). Compared with WT, 736 upregulated and 1,461 downregulated genes were identified in Δ*Chatf1* (Fig. S4B). Notably, in Δ*Chcpc1* and Δ*Chatf1* mutants, the number of downregulated genes was much greater than that of upregulated genes, indicating that *ChCPC1* and *ChATF1* mainly had positive regulatory effects on gene expression. Comparison of DEGs showed that there was a high overlap (*n* = 821) between DEGs of Δ*Chcpc1* (*n* = 1,541) and those of Δ*Chatf1* (*n* = 2197). Among the 821 overlapped DEGs, 245 DEGs were upregulated, and 563 DEGs were downregulated (Fig. S4C and D).

To investigate the transcriptomic changes associated with the absence of *ChCPC1* or *ChATF1*, we used Gene Ontology (GO) classification to determine the functions of DEGs. The GO enrichment analysis revealed that several biological processes were enriched among the DEGs of the Δ*Chcpc1* and Δ*Chatf1* mutants, which included intrinsic component of membrane, integral component of membrane, carbohydrate metabolic process, and transmembrane transporter activity; cofactor binding was also enriched among the DEGs of the Δ*Chcpc1* mutant (Fig. S4E and F). Based on GO functional annotation, the overlapped upregulated DEGs between Δ*Chcpc1* and WT and between Δ*Chatf1* and WT focused mainly on lipid metabolic process, proteolysis, nucleus, intracellular membrane-bounded organelle, and membrane-bounded organelle (Fig. S5A). The overlapped downregulated DEGs were enriched mainly in the oxoacid metabolic process, organic acid metabolic process, lipid metabolic process, nucleus, intracellular membrane-bounded organelle, and membrane-bounded organelle (Fig. S5B).

In addition to determining the physiological processes controlled by ChCpc1 or ChAtf1, we performed a Kyoto Encyclopedia of Genes and Genomes (KEGG) enrichment analysis. The top KEGG pathway enrichment analysis revealed that DEGs were involved mainly in the biosynthesis of secondary metabolites, carbon metabolism, valine, leucine and isoleucine degradation, and biosynthesis of amino acid in the Δ*Chcpc1* mutant (Fig. S4G). However, in the Δ*Chatf1* mutant, the most enriched pathway was biosynthesis of secondary metabolites, followed by amino sugar and nucleotide sugar metabolism, tryptophan metabolism, and starch and sucrose metabolism (Fig. S4H). The overlapped upregulated DEGs between Δ*Chcpc1* and WT and between Δ*Chatf1* and WT were highly clustered in biosynthesis of secondary metabolites, carbon metabolism, and glyoxylate and dicarboxylate metabolism (Fig. S5C). The KEGG pathway enrichment analysis showed that overlapped downregulated DEGs were involved in biosynthesis of secondary metabolites, biosynthesis of cofactors, and pentose and glucuronate interconversions pathways (Fig. S5D).

## DISCUSSION

New approaches into the molecular pathways that regulate plant infection by fungal pathogens could include investigating attractive targets to manage a series of plant diseases effectively. Nutrient utilization is a key factor during conidiation and the infection process in fungal pathogens, but little is known about the regulatory mechanism of nitrogen and amino acid metabolism, which limits the exploration of novel intervention strategies.

Here, we clarified nitrogen and amino acid metabolism involved in conidiation and the infection process of *C. heterostrophus*, and we showed how ChCpc1 coordinated amino acid synthesis and autophagy induction to promote conidiation and infection. ChCpc1 is a bZIP transcription factor, and bZIP TFs are highly conserved; they play essential roles in regulating transcription of genes involved in development, infection, and response to environmental changes in eukaryotes (18–20, 25). MoAp1 and MoAtf1 were identified as bZIP TFs in *M. oryzae* and are critical in conidiogenesis and pathogenicity. Subsequently, the functions of 19 additional bZIP TFs were characterized, and most of these TFs functioned actively in conidiogenesis (25). The bZIP transcription factor VdMRTF1 is a negative regulator of melanin biosynthesis and virulence, but VdAtf1 regulated virulence by mediating nitrogen metabolism in *V. dahliae* (20, 26). In addition, the bZIP transcription factor AfRsmA was also required for aflatoxin B1 biosynthesis, oxidative stress response, and sclerotium formation in *Aspergillus flavus* (27). General control non-derepressible 4 (GCN4), which is a key transcription factor that responds to nitrogen metabolism, bound to the GIMPC promoter region and participated in regulating the tricarboxylic acid cycle and ganoderic acid biosynthesis under low nitrogen conditions in *Ganoderma lucidum* (28). Cpc1 is a homologue of the bZIP transcription factor Gcn4 in yeast, and to the best of our knowledge, our study is the first to report on the regulatory mechanism of Cpc1 response to nitrogen deficiency in a plant fungal pathogen.

*C. heterostrophus* is a destructive pathogen of corn and a representative of the largest taxonomic group of fungal plant pathogens, the Dothideomycetes. The asexual conidia stage is very important in the life cycle of *C. heterostrophus* because dispersal in the field mostly depends on asexual conidia. After the conidiation or conidial germination was blocked, the disease did not spread in the field. To analyze the transcriptional changes of *C. heterostrophus* during the infection process, we conducted the RNA-seq analysis using the *C. heterostrophus* inoculated detached maize leaves (sampled at 12 hpi and 24 hpi). The transcriptomic results revealed that several pathways for amino acid metabolism were enriched during conidiation and the infection process, and this demonstrated that amino acid synthesis may play a crucial role in *C. heterostrophus* (24). Until now, there have been few reports on the involvement of amino acid synthesis in development, sporulation, and pathogenicity in fungi. In *M. oryzae*, leucine and asparagine biosynthesis were required for vegetative growth, conidiation, and full virulence (11, 12, 29, 30). Similar results were also found in *V. dahliae* and *V. longisporum* (13, 22). However, how the amino acid synthesis genes were regulated and how they were ordinated during conidiation and the infection process in plant pathogenic fungi have been reported seldomly. In fungal models that used *Neurospora crassa* and *Aspergillus nidulans*, amino acid starvation led to Cpc1-mediated activation of amino acid biosynthesis genes (31–33). To date, reports in some plant pathogenic fungi also demonstrated the relationship between Cpc1 and amino acid synthesis under amino acid starvation (4, 21, 22). However, the strong link between Cpc1 regulation on amino acid synthesis has not been reported in other plant pathogenic fungi, and the detailed regulatory mechanism was never investigated. It certainly will provide fascinating insights into the transcriptional mechanisms on amino acid regulation in phytopathogenic fungi by expanding current findings into other species of Dothideomycetes.

In our study, ChCpc1 activated the expression of a series of *ATG* genes by binding to the promoter region to maintain the conidiation and pathogenicity in *C. heterostrophus*. Li *et al*. showed that biotrophic growth of *M. oryzae* required cycles of Rim15-coordinated autophagy and glutaminolysis to yield α-ketoglutarate, which is a cell-intrinsic nutrient sufficiency signal that reactivated TOR and fuels growth while conserving glucose for antioxidation-mediated host defense suppression (10). In addition, autophagy contributed to the regulation of nuclear dynamics during vegetative growth and hyphal fusion in *Fusarium oxysporum* (15). Csn5, which is subunit 5 of COP9 signalosome (CSN), inhibited autophagy by regulating the ubiquitination of Atg6 and Tor to mediate the pathogenicity of *M. oryzae* (34). Several genes were related to autophagy in *M. oryzae*; for example, MoVast2 interacted with MoVast1 and MoAtg8 to regulate autophagy (35), MoAtg1-dependent MoMkk1 phosphorylation was essential for the pathogenicity of *M. oryzae* (16), and histone acetyltransferase MoHat1 acetylated MoAtg3 and MoAtg9 to orchestrate functional appressorium formation and pathogenicity in *M. oryzae* (36). All these results proved the important role of autophagy in development and pathogenicity in plant fungal pathogens. In accordance with these results, our study demonstrated the essential roles of *ChATG* genes during conidiation and the infection process, and it clarified the regulatory mechanism of ChCpc1 on *ChATG* genes, especially under amino acid limitation conditions.

Atf1, which is a bZIP transcription factor, is a target of the mitogen-activated protein kinase that regulated cell integrity and sensitivity to azole antifungals in fission yeast (37, 38). In addition, Atf1 promoted stress tolerance under nitrosative stress in *Schizosaccharomyces pombe* (39). Atf1 is also essential for full virulence, toxin production, and stress tolerance in plant fungal pathogens. CsAtf1 was involved in virulence and fludioxonil sensitivity in the rubber tree anthracnose fungus *Colletotrichum siamense* (40). In the cereal pathogen *F. graminearum*, Atf1 was essential for full virulence, deoxynivalenol production, and stress tolerance (19). Deletion of *FvATFA*, which is an orthologous gene of *ATF1*, resulted in subnormal vegetative and invasive growth, increased sensitivity to abiotic stress, decreased mycotoxin, and decreased pigment production in *Fusarium verticillioides* (41). In addition, VdAtf1 governed pathogenesis through the regulation of nitrosative resistance and nitrogen metabolism in *V. dahliae* (20). In our study, ChAtf1 responded to amino acid starvation and interacted with ChCpc1 to promote the transcriptions of *ChARG4* and *ChATG8*. This result proved that ChAtf1 may form a heterodimer with ChCpc1 to enhance the binding of ChCpc1 to the promoter regions of *ChARG4* and *ChATG8*. However, unlike in the plant fungal pathogens mentioned above, deletion of *ChATF1* had no effect on the virulence of *C. heterostrophus*. Therefore, we speculate that Atf1 may play different roles in virulence in different fungal pathogens. However, after at least 10 times of transformations, we still could not obtain the GFP-ChCpc1 strain. We suspect that the expression level of ChCpc1 is very low, which may have prevented us from obtaining the GFP-ChCpc1 strain. Additionally, we also attempted to prepare a specific antibody for ChCpc1 but were unsuccessful. Therefore, in this study, we did not validate the interaction between ChCpc1 and ChAtf1 through *in vivo* experiments. In the future, we will try more methods for *in vivo* validation.

The mitogen-activated protein kinase encoding gene *ChCHK1*, which is homologous to the yeast *FUS3/KSS1*, was involved in conidiation, appressorium formation, and pathogenicity in *C. heterostrophus* (42). In addition, the influence of Chk1 on the expression of melanin biosynthesis genes through the *C. heterostrophus* Cmr1 ortholog was revealed by Eliahu et al. (43). In our study, ChChk1 was not only involved in conidiation and pathogenicity, it was also necessary for adaptation to amino acid starvation. In addition, we revealed the association between the mitogen-activated protein kinase ChChk1 and transcription factor ChCpc1. In the future, we will investigate the regulatory mechanism of ChChk1 on ChCpc1.

## MATERIALS AND METHODS

### Fungal strains and cultural condition

The wild-type strain C4 (*Tox1*^+^, *MAT1-2*, ATCC48331) of *C. heterostrophus* was used as a parental strain and cultured on complete medium with xylose (CMX) at 25°C (14). For special purposes, mycelial plugs were inoculated on MM [1 g Ca(NO_3_)_2_·4H_2_O, 0.2 g KH_2_PO4, 0.25 g MgSO_4_·7H_2_O, 0.15 g NaCl, 10 g glucose, and 20 g agar in 1 L of double-distilled water] supplemented with indicated chemicals. The construction of GFP-ChAtg8, autophagy induction and monitoring, and western blot analysis were conducted as previously described (14). Yeast nitrogen base with amino acids (YNB-AA), ammonium sulfate (5 g/L), biotin (2 µg/L), calcium pantothenate (400 µg/L), folic acid (2 µg/L), inositol (2,000 µg/L), nicotinic acid (400 µg/L), p-aminobenzoic acid (200 µg/L), pyridoxine HCl (400 µg/L), riboflavin (200 µg/L), thiamine HCl (400 µg/L), boric acid (500 µg/L), copper sulfate (40 µg/L), potassium iodide (100 µg/L), ferric chloride (200 µg/L), manganese sulfate (400 µg/L), sodium molybdate (200 µg/L), zinc sulfate (400 µg/L), potassium phosphate monobasic (1 g/L), magnesium sulfate (0.5 g/L), sodium chloride (0.1 g/L), and calcium chloride (0.1 g/L) (final pH: 5.4 ± 0.2 at 25°C) purchased from Sigma (291920) was used to simulate amino acid starvation conditions (44).

### *C. heterostrophus* mutant generation

The polyethylene glycol (PEG)-mediated protoplast transformation method was used for gene deletion in *C. heterostrophus* (24). In brief, fresh protoplasts of *C. heterostrophus* were made from fresh mycelia treated with driselase and glucanex. Flanking and *HPH* fragments were amplified with indicated primers (Table S2). Hygromycin was used as the selection marker, and mutant candidates were verified by diagnostic PCR (Table S2). For complementation, the WT target gene and its 5′ and 3′ flanking regions, the *NPTII* gene, and a fragment further 3′ of the target gene 3′ flanking region were amplified. Then, the three fragments were used to transform the mutant, and transformants were screened on CMX plates amended with geneticin and verified by PCR (24).

### Phenotypic analysis

To determine the mycelial growth, 5 mm mycelial plugs of each strain were inoculated on CMX, and the colony diameters of each strain were measured and recorded. For the conidiation assay, the same area of 7-day-old cultures on CMX from each strain was harvested to dislodge conidia and filtered with four layers of cheesecloth to remove mycelial debris. Conidia were counted using a hemocytometer. Melanin extraction and determination were conducted as described previously (45). In brief, WT, Δ*cpc1* mutant, and the complemented strain were cultured in liquid CMX for 48 h; dried mycelia were ground in liquid nitrogen and dissolved in NaOH. After centrifugation, the supernatant was adjusted to pH 2.0 with HCl, and the pellet was dissolved in NaOH. The concentration of melanin was measured using ultraviolet spectrophotometry.

For amino acid concentration determination, liquid CM (D-glucose (10 g/L), yeast extract (1 g/L), casamino acids (1 g/L), calcium nitrate tetrahydrate (1 g/L), potassium dihydrogen phosphate (0.2 g/L), magnesium sulfate heptahydrate (0.2 g/L), sodium chloride (0.15 g/L), boric acid (28.6 µg/L), copper sulfate pentahydrate (196.5 µg/L), potassium iodide (6.55 µg/L), manganese sulfate monohydrate (30.2 µg/L), ammonium heptamolybdate tetrahydrate (18.4 µg/L), zinc sulfate monohydrate (2.745 mg/L), and iron chloride hexahydrate (474.1 µg/L) (final pH: 6.5 ± 0.2 at 25°C) was inoculated with mycelia. After 36 h of shaking at 25°C, the mycelia were transferred into MM or MM supplemented with 100 µM MSX. After another 2 h of shaking at 25°C, fresh mycelia were used for the amino acid concentration determination. The total amino acid concentration was determined using the Amino Acid (AA) Content Assay Kit purchased from Beijing Boxbio Science & Technology Co., Ltd. The arginine concentration was determined using the Arginine (Arg) Content Assay Kit purchased from Beijing Solarbio Science & Technology Co., Ltd.

### Virulence test

Three-week-old maize (*Z. mays* B73) leaves were used for virulence testing. The mycelial plugs of WT or mutants harvested from 3-day-old cultures were inoculated on the detached maize leaves. The inoculated maize leaves were transferred to a petri dish and kept for 24 h; they were then moved out and kept at 25°C under 16 h of light/8 h of dark. After 3 days, the lesion size was measured with ImageJ. The virulence test was repeated at least three times, and three replications were conducted each time.

### Yeast one-hybrid (Y1H)

For Y1H analysis, the full-length coding sequence of *ChCPC1* was amplified and constructed into the pGADT7 vector. The DNA fragments that contained promoter regions of target genes were inserted into the reporter vector pAbAi to generate the pAbAi-bait plasmids. The recombinant pGADT7-Cpc1 plasmid was used to transform Y1HGold yeast competent cells that carried the linearized pAbAi-bait. DNA-protein interactions were detected by the growth of serial dilutions of transformants on nutrient-deficient medium supplemented with 200 µg/mL aureobasidin A (AbA).

### Yeast two hybrid (Y2H)

Y2H was used to verify the protein-protein interaction. The coding sequence of the indicated gene that was amplified from cDNA of C4 with corresponding primers was cloned into pGBKT7 or pGADT7 to construct bait or prey plasmids. Both bait and prey vectors were verified by sequencing and co-transformed into the yeast strain AH109 following the lithium acetate method. After growing on synthetic medium (SD) that lacked Leu and Trp, the serially diluted yeast cells were transferred to SD without Leu, Trp, His, and Ade to confirm the protein-protein interaction. The interaction between pGADT7-T and pGBKT7-53 was used as a positive control, while that between pGADT7-T and pGBKT7-Lam was used as a negative control.

### Yeast two-hybrid library screening

To explore the interacting proteins of ChCpc1, a yeast two-hybrid (Y2H) library screening was performed. Using ChCpc1-BD as bait, we screened a cDNA activator library of *C. heterostrophus* constructed by OE Biotech Co., Ltd. (Shanghai, China). Then, the potential interactors were identified by gene sequencing.

### EMSA

The binding motif of ChCpc1 was obtained by blasting with JASPAR (http://jaspar.genereg.net) (46). The coding sequence of the protein ChCpc1 was amplified and cloned into the pET-32a to generate a His-tagged protein. Then, ChCpc1-His was purified with Ni Sepharose beads and eluted with imidazole. The Light Shift Chemiluminescent EMSA Kit (Beyotime, Shanghai, China) was used for the EMSA assay following the manufacturer’s instructions. The recombinant Cpc1 proteins were incubated with biotin-labeled probes for 20 min at 25°C. Competitive probes (100×) were used for competition experiments. The above mixed reactions were separated using 6.5% polyacrylamide gels with a running buffer of 0.5× Tris-borate-EDTA, and then they were transferred to a positively charged nylon membrane. Signals were detected using the chemiluminescent substrate in the kit according to the manufacturer’s instructions. Images were acquired using a charge-coupled device (CCD) camera.

### GST pull-down assay

To perform GST pull-down assays, the full-length of ChCpc1 was amplified from the cDNA of C4 and cloned into pGEX-6P-1 to generate GST-tagged plasmids. In addition, the full length of ChAtf1 was amplified and cloned into pET32a to generate His-tagged plasmids. GST- and His-tagged plasmids were expressed in *Escherichia coli* BL21 and purified. To verify the interaction between GST- and His-tagged proteins, GST-tagged proteins were mixed with GST Sepharose beads and incubated for 4 h at 4°C; then they were mixed with His-tagged proteins for another 4 h at 4°C. The beads were washed five times with TBS buffer, and the proteins were eluted. GST was used as a negative control. Finally, the protein interactions were determined by anti-His and anti-GST antibodies.

### Endogenous ChAtg8 detection

For endogenous ChAtg8 detection, the WT C4 and Δ*Chcpc1* mutant strains were grown in liquid CM medium at 25°C for 36 h. Then, the mycelia were introduced into MM or MM supplemented with 100 µM MSX for 2 h. Proteins were extracted with lysis buffer (Beyotime Biotechnology, China, product number: P0013) and analyzed by Western blotting. ChAtg8 was detected with Anti-Atg8 (Filamentous fungi) pAb (MBL Life Science, Japan).

### Metabolomic analysis

Mycelial tissues of WT and Δ*Chcpc1* mutant strains cultured on MM or MM supplemented with MSX were collected and ground with liquid nitrogen. The homogenate was resuspended in 80% methanol and centrifuged at 15,000 × *g* at 4°C for 20 min. Finally, the supernatant was injected into an LC-MS/MS system for analysis.

UHPLC-MS/MS analyses were performed using the Vanquish UHPLC System (Thermo Fisher, Germany) coupled with an Orbitrap Q ExactiveTM HF mass spectrometer by Novogene Co., Ltd. (Beijing, China). The samples were analyzed using a Hypersil GOLD column (100 × 2.1 mm, 1.9 µm) with a 12 min linear gradient at a flow rate of 0.2 mL/min. The eluents for the positive and negative polarity modes were eluents A (0.1% FA in water) and B (methanol). The solvent gradient was set as follows: 2% B, 1.5 min; 2–85% B, 3 min; 85–100% B, 10 min; 100–2% B, 10.1 min; and 2% B, 12 min. The Q Exactive HF mass spectrometer was operated in positive/negative polarity mode with a spray voltage of 3.5 kV, a capillary temperature of 320°C, a sheath gas flow rate of 35 psi, an aux gas flow rate of 10 L/min, S-lens RF level of 60, and an aux gas heater temperature of 350°C. The normalized data were used to predict the molecular formula based on additive ions, molecular ion peaks, and fragment ions. Peaks were matched with the mzCloud (https://www.mzcloud.org/), mzVault, and MassList databases to obtain accurate qualitative and relative quantitative results. Statistical analyses were performed using statistical software R (R version R-3.4.3), Python (Python version 2.7.6), and CentOS (CentOS release 6.6). The metabolites were annotated using the KEGG (https://www.genome.jp/kegg/pathway.html), HMDB (https://hmdb.ca/metabolites), and LIPIDMaps databases (http://www.lipidmaps.org/).

### RNA extraction and transcriptomic analysis

Liquid CMX medium was inoculated with 5 mL (1 × 10^4^ conidia/mL) of conidial suspension of WT, Δ*Chcpc1*, and Δ*Chatf1* mutants. After 24 h of shaking at 25°C, fresh mycelia were used for RNA extraction. Total RNA of WT, Δ*Chcpc1*, and Δ*Chatf1* mutants was extracted with Trizol reagent and evaluated with an RNA Nano 6000 Assay Kit. Transcriptomic analysis was conducted as described previously and performed by Novogene Technologies Corporation (Beijing, China). The reads were mapped to the reference genome of *C. heterostrophus* (https://mycocosm.jgi.doe.gov/CocheC4_6/CocheC4_6.home.html) using HISAT2 (v.2.1.0) with default settings. The mapped reads of each sample were assembled by StringTie (v.1.3.3b) using a reference-based approach. The FPKM of each gene was calculated based on the length of the gene and reads count mapped to this gene, and differential expression analysis was performed using the DESeq2 R package. A corrected *P*-value of 0.05 and absolute fold change of 2 were set as the threshold for a significantly differential expression. GO enrichment analysis of DEGs was performed using the R package clusterProfiler, and *P*  <  0.05 was considered significantly enriched regarding DEGs. We used the R package clusterProfiler to test the statistical enrichment of DEGs in the KEGG pathways.

### *In vitro* phosphorylation assay

In yeast and animals, MAPKKs are activated by phosphorylation of two serine/threonine residues by MAPKKKs in a conserved S/TXXXS/T motif. When these two Ser/Thr residues were replaced by Glu (E) or Asp (D), the point-mutated MAPKK became constitutively active (47–49). Therefore, we used the recombinant Ste7DD to activate downstream Chk1. To test whether ChChk1 can directly phosphorylate ChCpc1, the *in vitro* phosphorylation assay was conducted following the protocol described in the work of Cruz-Mireles et al. (47). In brief, 6× His tagged Chk1 was activated by incubation with Ste7^DD^ for 30 min in kinase buffer (1 mM DTT, 1 mM EGTA, 10 mM MnCl_2_, and 25 mM Tris pH 7.5) in the presence of 1 mM ATP at 30°C, then it was incubated with GST-tagged Cpc1 for another 30 min. Proteins were separated by SDS-PAGE and transferred to a polyvinylidene difluoride (PVDF) membrane. GST antibody (Sangon) and 6 × His antibody (Proteintech) were used to detect GST and His tags, respectively. Cpc1 phosphorylation was detected using pS/pT-P antibody (Cell Signaling). The Pierce ECL western blotting substrate (Thermo Fisher Scientific) was used for detection, and membrane images were acquired using a charge-coupled device (CCD) camera.

### Statistical analysis

For statistical analysis, GraphPad Prism program’s *t*-tests and Bonferroni correction of multiple *t*-tests were used for the analysis of significant differences. All data shown are presented as mean  ±  standard error of the mean (SEM).

## Data Availability

RNA-seq data are available at NCBI under accession no. PRJNA1247738.

## SUPPLEMENTAL MATERIAL

Supplemental figures mbio.00845-25-s0001.docx Fig. S1–S5.

Supplemental tables mbio.00845-25-s0002.docx Tables S1 and S2.

ASM does not own the copyrights to Supplemental Material that may be linked to, or accessed through, an article. The authors have granted ASM a non-exclusive, world-wide license to publish the Supplemental Material files. Please contact the corresponding author directly for reuse.
