# Supplementary material for: ChCpc1, a bZIP transcription factor, coordinates amino acid synthesis and autophagy and modulates conidiation and virulence in Cochliobolus heterostrophus
Source: mBio. 2025 Jul 21;16(8):e00845-25. doi: 10.1128/mbio.00845-25 (PMC12345223; doi:10.1128/mbio.00845-25)
Supplement: Supplemental figures — Fig. S1–S5. [file mbio.00845-25-s0001.docx]

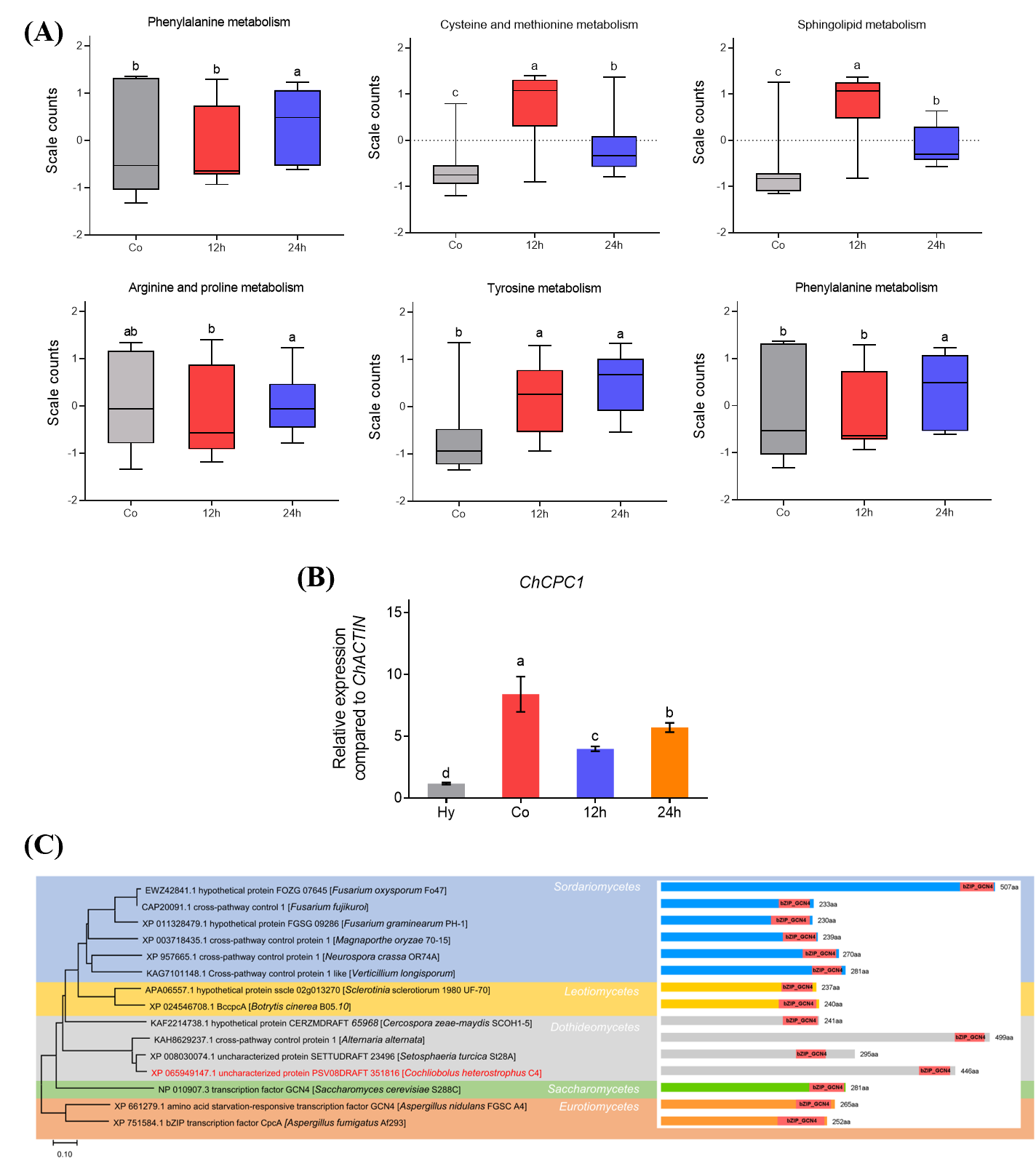


Fig. S1 Several pathways related to nitrogen and amino acid metabolism were enriched in conidiation and the infection process of *C. heterostrophus*. (A) The enrichment changes of indicated pathways at 12- and 24-hours post-inoculation (hpi). (B) Relative expression levels of *ChCPC1* during different developmental stages (Hy: hypha, Co: conidia, 12 h: 12 hours post-inoculation, 24 h: 24 hours post-inoculation). (C) Phylogenetic and molecular evolutionary analysis of Cpc1 in different organisms were conducted using MEGA X.


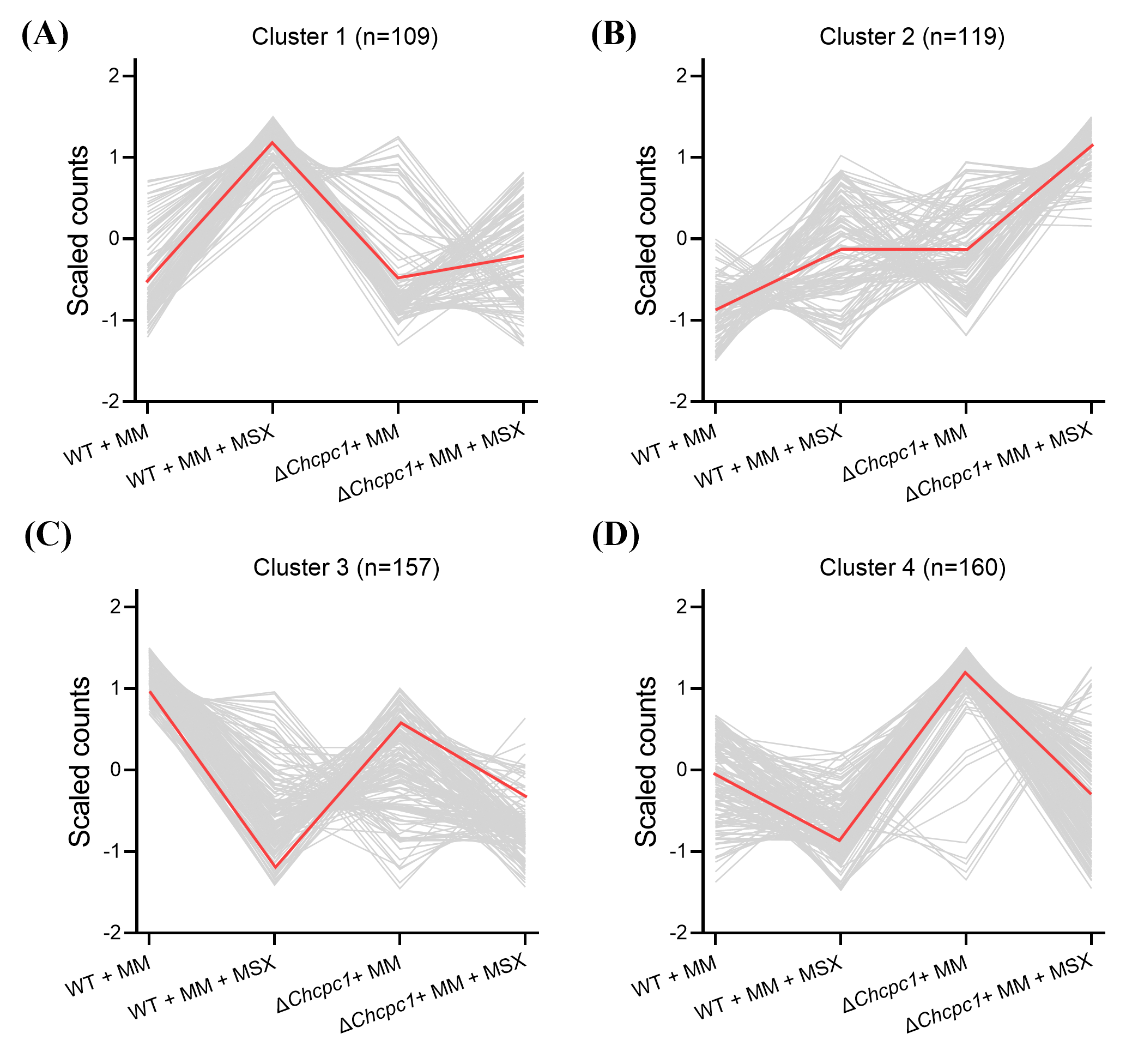


Fig. S2 K-means clustering analysis of differentially accumulated metabolites (DAMs) in WT and Δ*cpc1* mutants treated with L-Methionine-DL-sulfoximine (MSX). Four clusters (A, B, C, and D) were identified and variation trend of DAMs were different in each cluster. The vertical coordinates represent the normalized (Z-score) metabolite relative content. n: the numbers of metabolites in this cluster. MM: minimal medium; MM + MSX: minimal medium supplemented with L-Methionine-DL-sulfoximine (MSX).


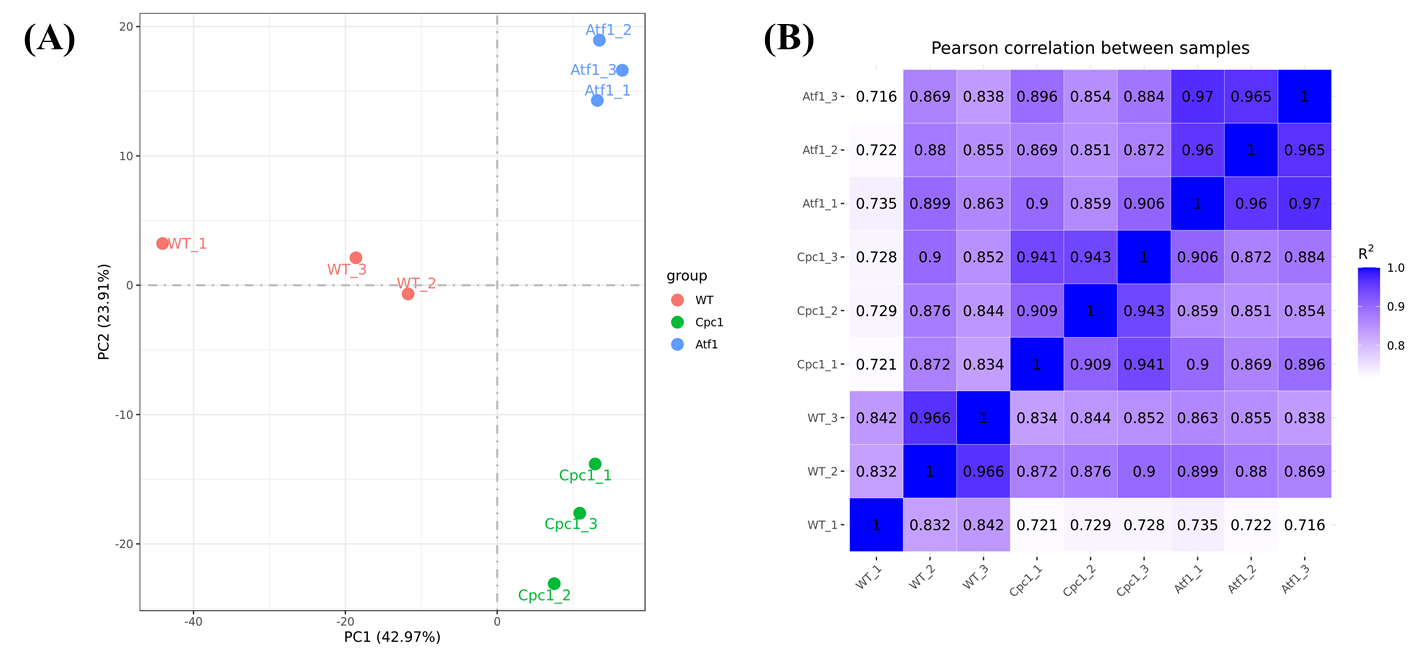


Fig. S3 Principal component analysis and Pearson correlation coefficient of the transcriptome data sets. (A) Principal component analysis (PCA) of the transcriptome data sets. (B) Pearson correlation coefficient of the transcriptome data sets.


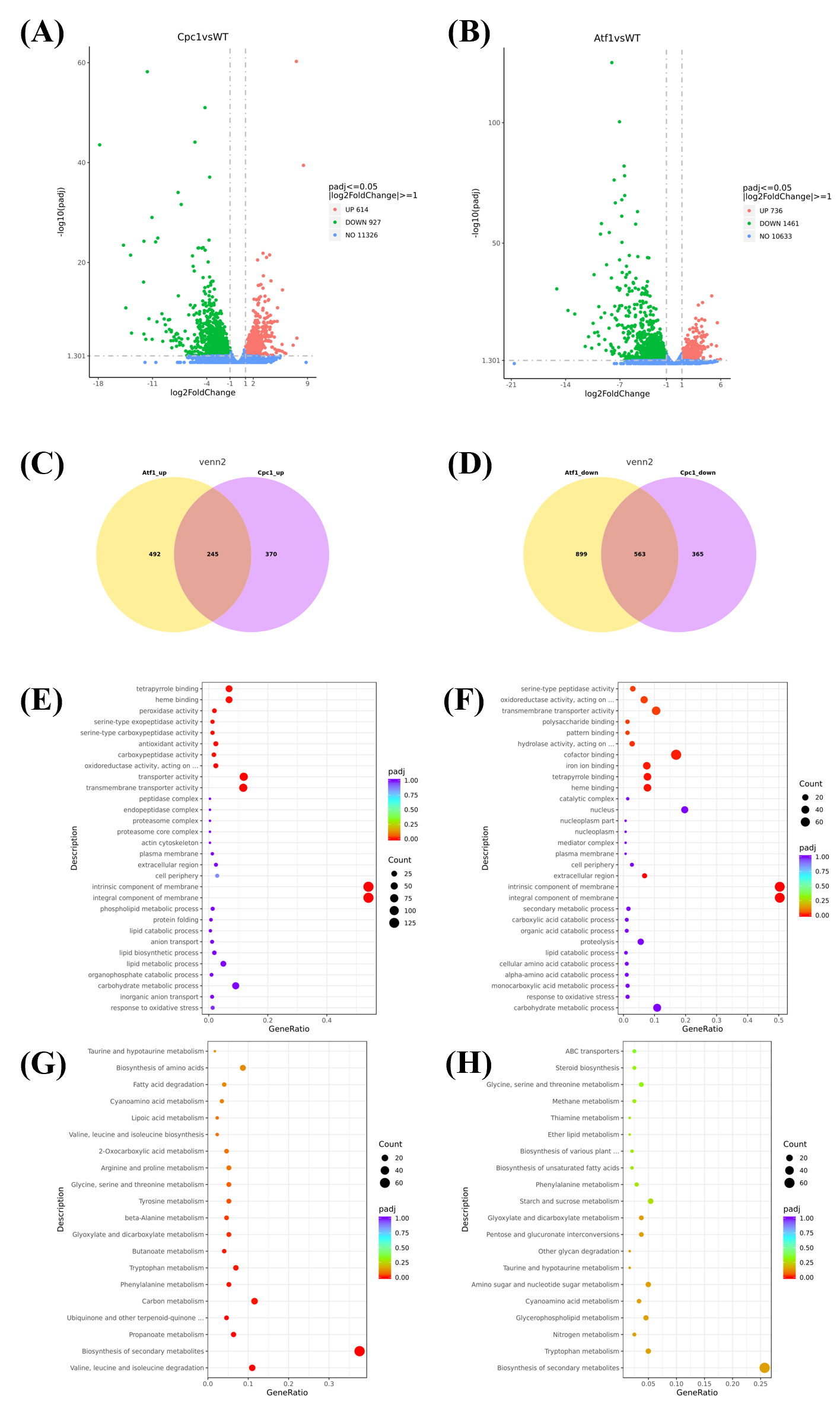


Fig. S4 Identifying *ChCPC1*-dependent and *ChATF1*-dependent changes in gene expression of *C. heterostrophus*. (A, B) Volcano plot of the distribution of differential expressed genes (DEGs) in Δ*Chcpc1* or Δ*Chatf1* compared to WT. The red colour and green colour represent the significantly up-regulated genes and down-regulated genes, respectively, while the blue colour indicates no significant expression. The x axis represents the change in gene expression in different samples, here represented by log_2_[fold change] value. The y axis indicates the statistical significance of changes in gene expression levels, here represented by −log_10_(*p*_adj_) value. (C) Venn diagram showing a comparison of up-regulated genes from two comparisons. (D) Venn diagram showing a comparison of down-regulated genes from two comparisons. (E, F) Gene ontology (GO) term enrichment analysis of DEGs in Δ*Chcpc1* and Δ*Chatf1*, respectively. (G, H) Kyoto Encyclopedia of Genes and Genomes (KEGG) enrichment analysis of DEGs in Δ*Chcpc1* and Δ*Chatf1*, respectively.


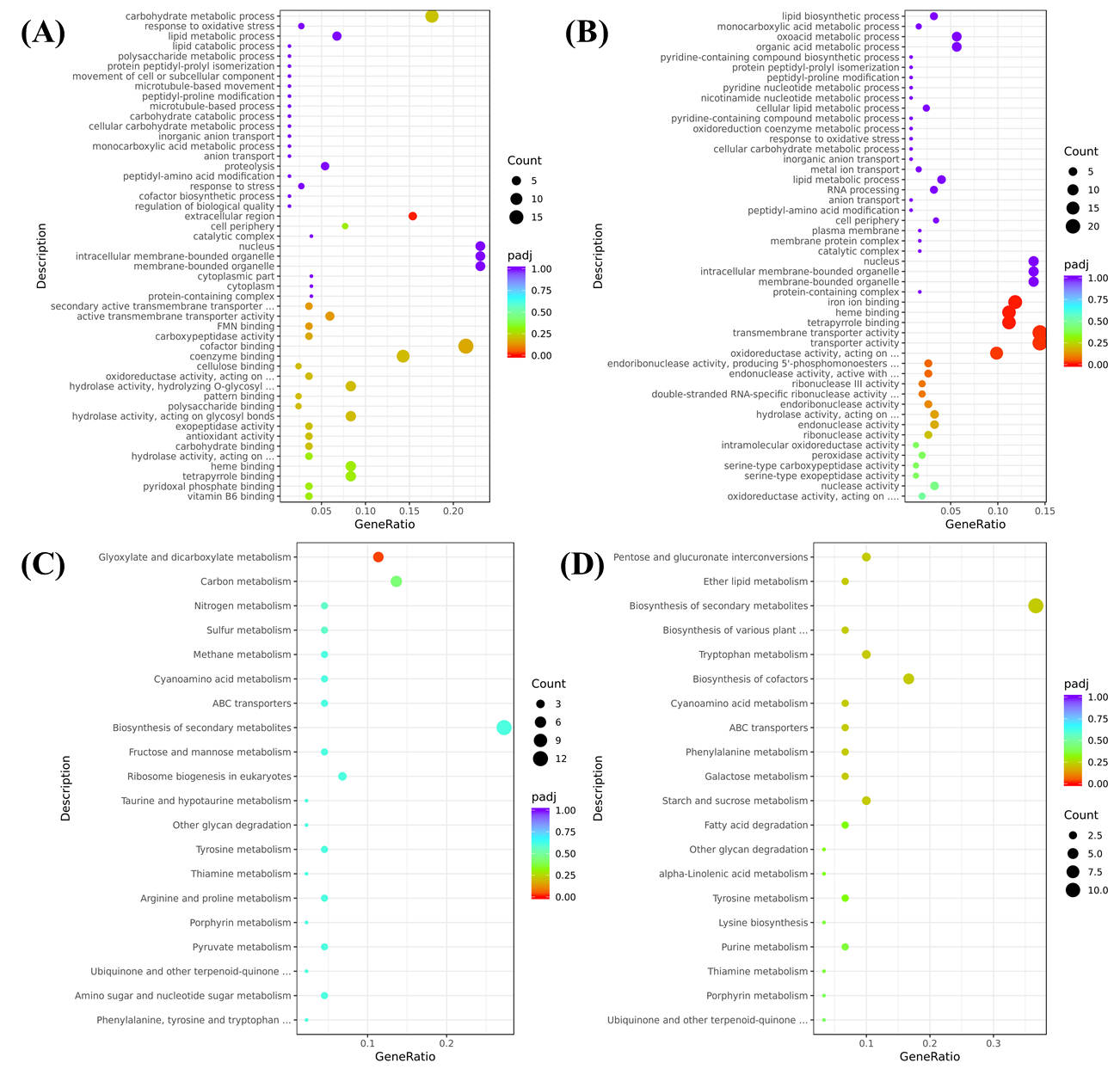


Fig. S5 Gene ontology (GO) term and Kyoto Encyclopedia of Genes and Genomes (KEGG) enrichment analysis of the overlapped DEGs between Δ*Chcpc1* and WT and between Δ*Chatf1* and WT. (A) Gene ontology (GO) term enrichment analysis of the overlapped up-regulated DEGs between Δ*Chcpc1* and WT and between Δ*Chatf1* and WT. (B) Gene ontology (GO) term enrichment analysis of the overlapped down-regulated DEGs between Δ*Chcpc1* and WT and between Δ*Chatf1* and WT. (C) Kyoto Encyclopedia of Genes and Genomes (KEGG) enrichment analysis of the overlapped up-regulated DEGs between Δ*Chcpc1* and WT and between Δ*Chatf1* and WT. (D) Kyoto Encyclopedia of Genes and Genomes (KEGG) enrichment analysis of the overlapped down-regulated DEGs between Δ*Chcpc1* and WT and between Δ*Chatf1* and WT.X
