# Supplementary material for: ChCpc1, a bZIP transcription factor, coordinates amino acid synthesis and autophagy and modulates conidiation and virulence in Cochliobolus heterostrophus
Source: mBio. 2025 Jul 21;16(8):e00845-25. doi: 10.1128/mbio.00845-25 (PMC12345223; doi:10.1128/mbio.00845-25)
Supplement: Supplemental tables — Tables S1 and S2. [file mbio.00845-25-s0002.docx]

Table S1 List of differential accumulated metabolites

| Compared samples | Numbers of total significant | Numbers of significant up-regulated | Numbers of significant down-regulated |
| --- | --- | --- | --- |
| WT_MM_MSX. vs. WT_MM | 209 | 105 | 104 |
| cpc1_MM_MSX. vs. cpc1_MM | 196 | 71 | 125 |
| cpc1_MM_MSX. vs. WT_MM_MSX | 120 | 75 | 45 |
| cpc1_MM. vs. WT_MM | 151 | 91 | 60 |

Table S2 Primers used in this study.

| **Primer** | **Sequence 5’ to 3’** | **Description** | **Purpose** |
| --- | --- | --- | --- |
| Chcpc1-FP1 | TGGCTCAGTGCCGTCGTGT | 5’ flanking region of *CPC1,* forward primer | Delete *CPC1* |
| Chcpc1-RP1 | TCCTGTGTGAAATTGTTATCCGCT  GCTGAAGCCACTTGAGATGA | 5’ flanking region of *CPC1,* reverse primer with hygB gene tail |  |
| Chcpc1-FP2 | GTCGTGACTGGGAAAACCCTGGCGCCCAGTGGTCGTTAGGAG | 3’ flanking region of *CPC1,* forward primer with hygB gene tail |  |
| Chcpc1-RP2 | AGGCGATAATATGAGGAGAA | 3’flanking region of *CPC1,* reverse primer |  |
| M13R | AGCGGATAACAATTTCACACAGGA | Forward primer to amplify hygB |  |
| M13F | CGCCAGGGTTTTCCCAGTCACGAC | Reverse primer to amplify hygB |  |
| NLC37 | GGATGCCTCCGCTCGAAGTA | Located at hygB, reverse primer | *CPC1* deletion verification |
| Chcpc1-UF | ACCCTTGACCTGGTGGCA | Upstream verification forward primer of *CPC1*, pair with NLC37 |  |
| NLC38 | CGTTGCAAGACCTGCCTGAA | Located at hygB, forward primer |  |
| Chcpc1-DR | CTTTGTGCCTGTAAGGGT | Downstream verification reverse primer of *CPC1*, pair with NLC38 |  |
| Chcpc1-F | CCGCCACCATACCCACTCT | Forward primer to amplify *CPC1* |  |
| Chcpc1-R | GAACCTTGGTCTCCTGCTC | Reverse primer to amplify *CPC1* |  |
| Chcpc1-C1 | CACTGGAACAACTGGCATGAGGCGATAATATGAGGAGAA | Complementation primer | *CPC1* complementation verification |
| Chcpc1-C2 | CAGGTACACTTGTTTAGATTCGGGTCCTTGATTAG |  |  |
| Chcpc1-C3 | GATGGCATAGTTGCTCGT |  |  |
| Chatf1-FP1 | GAGGGCGGCAGTGTTTGA | 5’ flanking region of *ATF1* forward primer | Delete *ATF1* |
| Chatf1-RP1 | TCCTGTGTGAAATTGTTATCCGCT  GCTCTGGCTGGCGATTGA | 5’ flanking region of *ATF1,* reverse primer with hygB gene tail |  |
| Chatf1-FP2 | GTCGTGACTGGGAAAACCCTGGCGGTAATCTCCGCACCTTCTC | 3’ flanking region of *ATF1,* forward primer with hygB gene tail |  |
| Chatf1-RP2 | CCTCCGCTCAATACCAAC | 3’flanking region of *ATF1,* reverse primer |  |
| M13R | AGCGGATAACAATTTCACACAGGA | Forward primer to amplify hygB |  |
| M13F | CGCCAGGGTTTTCCCAGTCACGAC | Reverse primer to amplify hygB |  |
| NLC37 | GGATGCCTCCGCTCGAAGTA | Located at hygB, reverse primer | *ATF1* deletion verification |
| Chatf1-UF | CAGAAAGGGAGCCGTTGG | Upstream verification forward primer of *ATF1*, pair with NLC37 |  |
| NLC38 | CGTTGCAAGACCTGCCTGAA | Located at hygB, forward primer |  |
| Chatf1-DR | GAAACAAACGCCCTCAAA | Downstream verification reverse primer of *ATF1*, pair with NLC38 |  |
| Chatf1-F | GTCACCCTCACCTTTCCC | Forward primer to amplify *ATF1* |  |
| Chatf1-R | TTGGCGATCCATTACAGC | Reverse primer to amplify *ATF1* |  |
| Chatg5-FP1 | TTATGGGTCCAGAGTTCG | 5’ flanking region of *ATG5,* forward primer | Delete *ATG5* |
| Chatg5-RP1 | TCCTGTGTGAAATTGTTATCCGCTGACAGCGTAGATGGGTGA | 5’ flanking region of *ATG5,* reverse primer with hygB gene tail |  |
| Chatg5-FP2 | GTCGTGACTGGGAAAACCCTGGCGCGAGTCGCAGTCAAGACG | 3’ flanking region of *ATG5,* forward primer with hygB gene tail |  |
| Chatg5-RP2 | TGTTAGGGATTGTGGGATGA | 3’flanking region of *ATG5,* reverse primer |  |
| M13R | AGCGGATAACAATTTCACACAGGA | Forward primer to amplify hygB |  |
| M13F | CGCCAGGGTTTTCCCAGTCACGAC | Reverse primer to amplify hygB |  |
| NLC37 | GGATGCCTCCGCTCGAAGTA | Located at hygB, reverse primer | *ATG5* deletion verification |
| Chatg5-UF | TAGTGGCGTTGGAAGGTG | Upstream verification forward primer of *ATG5*, pair with NLC37 |  |
| NLC38 | CGTTGCAAGACCTGCCTGAA | Located at hygB, forward primer |  |
| Chatg5-DR | GCAATTCGGTTGAGTCCC | Downstream verification reverse primer of *ATG5*, pair with NLC38 |  |
| Chatg5-F | CACCTGGAACGGGAGCAT | Forward primer to amplify *ATG5* |  |
| Chatg5-R | GCGGCACGTACCAAATCC | Reverse primer to amplify *ATG5* |  |
| Chatg7-FP1 | AGTGGTGCTGTTGAGGAGG | 5’ flanking region of *ATG7,* forward primer | Delete *ATG7* |
| Chatg7-RP1 | TCCTGTGTGAAATTGTTATCCGCTCAGTGTCTATGGCGTGTAGG | 5’ flanking region of *ATG7,* reverse primer with hygB gene tail |  |
| Chatg7-FP2 | GTCGTGACTGGGAAAACCCTGGCGTCGGCTCCATCGGTAAAT | 3’ flanking region of *ATG7,* forward primer with hygB gene tail |  |
| Chatg7-RP2 | CGAAGACGACGAACTCCA | 3’flanking region of *ATG7,* reverse primer |  |
| M13R | AGCGGATAACAATTTCACACAGGA | Forward primer to amplify hygB |  |
| M13F | CGCCAGGGTTTTCCCAGTCACGAC | Reverse primer to amplify hygB |  |
| NLC37 | GGATGCCTCCGCTCGAAGTA | Located at hygB, reverse primer | *ATG7* deletion verification |
| Chatg7-UF | TGAAGACAGAGGCAGGTGG | Upstream verification forward primer of *ATG7*, pair with NLC37 |  |
| NLC38 | CGTTGCAAGACCTGCCTGAA | Located at hygB, forward primer |  |
| Chatg7-DR | CGTCCTACAAGTCGCTTGATC | Downstream verification reverse primer of *ATG7*, pair with NLC38 |  |
| Chatg7-F | GCCCAGATTCTTTGTTACCG | Forward primer to amplify *ATG7* |  |
| Chatg7-R | CACCCTAGCTCTACCTCGTT | Reverse primer to amplify *ATG7* |  |
| Chatg8-FP1 | ATCTTCGGTGGAGTTTCGT | 5’ flanking region of *ATG8,* forward primer | Delete *ATG8* |
| Chatg8-RP1 | TCCTGTGTGAAATTGTTATCCGCTTCGTGGGAGTGGTGAGG | 5’ flanking region of *ATG8,* reverse primer with hygB gene tail |  |
| Chatg8-FP2 | GTCGTGACTGGGAAAACCCTGGCGTTGATTTGATGGCAGACG | 3’ flanking region of *ATG8,* forward primer with hygB gene tail |  |
| Chatg8-RP2 | GAGGGAGGCATACTTTGATA | 3’flanking region of *ATG8,* reverse primer |  |
| M13R | AGCGGATAACAATTTCACACAGGA | Forward primer to amplify hygB |  |
| M13F | CGCCAGGGTTTTCCCAGTCACGAC | Reverse primer to amplify hygB |  |
| NLC37 | GGATGCCTCCGCTCGAAGTA | Located at hygB, reverse primer | *ATG8* deletion verification |
| Chatg8-UF | CTCGGTGCTGACTTCTCGGG | Upstream verification forward primer of *ATG8*, pair with NLC37 |  |
| NLC38 | CGTTGCAAGACCTGCCTGAA | Located at hygB, forward primer |  |
| Chatg8-DR | GTTAGTATGTGTGAGCGG | Downstream verification reverse primer of *ATG8*, pair with NLC38 |  |
| Chatg 8-F | AGTTCAAGGACGAGCACC | Forward primer to amplify *ATG8* |  |
| Chatg8-R | TGGCAGGACCTCATCGACGAAG | Reverse primer to amplify *ATG8* |  |
| Chatg9-FP1 | ACGGGCTCCACGGTAGAAG | 5’ flanking region of *ATG9,* forward primer | Delete *ATG9* |
| Chatg9-RP1 | TCCTGTGTGAAATTGTTATCCGCTGCTGGATGGGTGCGTCTT | 5’ flanking region of *ATG9,* reverse primer with hygB gene tail |  |
| Chatg9-FP2 | GTCGTGACTGGGAAAACCCTGGCGTTTCCCTTATTCCCACTCGTAAAT | 3’ flanking region of *ATG9,* forward primer with hygB gene tail |  |
| Chatg9-RP2 | CTGGTGCTGATAGATTGC | 3’flanking region of *ATG9,* reverse primer |  |
| M13R | AGCGGATAACAATTTCACACAGGA | Forward primer to amplify hygB |  |
| M13F | CGCCAGGGTTTTCCCAGTCACGAC | Reverse primer to amplify hygB |  |
| NLC37 | GGATGCCTCCGCTCGAAGTA | Located at hygB, reverse primer | *ATG9* deletion verification |
| Chatg9-UF | TTCAGTCCCAAGAAGTACC | Upstream verification forward primer of *ATG9*, pair with NLC37 |  |
| NLC38 | CGTTGCAAGACCTGCCTGAA | Located at hygB, forward primer |  |
| Chatg9-DR | TCAACAAGATGTTGTCGTG | Downstream verification reverse primer of *ATG9*, pair with NLC38 |  |
| Chatg9-F | TCTCCAGATGCCAAACGT | Forward primer to amplify *ATG9* |  |
| Chatg9-R | AGCCGAGAAGGGTAAACA | Reverse primer to amplify *ATG9* |  |
| Charg1-FP1 | CTTCAAAGGCTGGACATAG | 5’ flanking region of *ARG1,* forward primer | Delete *ARG1* |
| Charg1-RP1 | TCCTGTGTGAAATTGTTATCCGCTATTCTTCTGACGGGTATCA | 5’ flanking region of *ARG1,* reverse primer with hygB gene tail |  |
| Charg1-FP2 | GTCGTGACTGGGAAAACCCTGGCGTTGGAAAGGGTCACTGGA | 3’ flanking region of *ARG1,* forward primer with hygB gene tail |  |
| Charg1-RP2 | GAGTCATGGGAGGCAGGT | 3’flanking region of *ARG1,* reverse primer |  |
| M13R | AGCGGATAACAATTTCACACAGGA | Forward primer to amplify hygB |  |
| M13F | CGCCAGGGTTTTCCCAGTCACGAC | Reverse primer to amplify hygB |  |
| NLC37 | GGATGCCTCCGCTCGAAGTA | Located at hygB, reverse primer | *ARG1* deletion verification |
| Charg1-UF | GCGACCTACGCCAATGCT | Upstream verification forward primer of *ARG1*, pair with NLC37 |  |
| NLC38 | CGTTGCAAGACCTGCCTGAA | Located at hygB, forward primer |  |
| Charg1-DR | CCTTCCCTTACCATTTATC | Downstream verification reverse primer of *ARG1*, pair with NLC38 |  |
| Charg1-F | TCCTACGAGGGCCGATAC | Forward primer to amplify*ARG1* |  |
| Charg1-R | CCATTGGGCTAAAGTTGTC | Reverse primer to amplify *ARG1* |  |
| Charg4-FP1 | CTCTTCTGATCGGTTTGA | 5’ flanking region of *ARG4,* forward primer | Delete *ARG4* |
| Charg4-RP1 | TCCTGTGTGAAATTGTTATCCGCT  TAGCAGGTCTGGATACTTTA | 5’ flanking region of *ARG4,* reverse primer with hygB gene tail |  |
| Charg4-FP2 | GTCGTGACTGGGAAAACCCTGGCGTTCCACGGTAAACATTCG | 3’ flanking region of *ARG4,* forward primer with hygB gene tail |  |
| Charg4-RP2 | AAACACCACCACCTCATCT | 3’flanking region of *ARG4,* reverse primer |  |
| M13R | AGCGGATAACAATTTCACACAGGA | Forward primer to amplify hygB |  |
| M13F | CGCCAGGGTTTTCCCAGTCACGAC | Reverse primer to amplify hygB |  |
| NLC37 | GGATGCCTCCGCTCGAAGTA | Located at hygB, reverse primer | *ATF1* deletion verification |
| Charg4-UF | TCACCGACTCGCCAGACA | Upstream verification forward primer of *ARG4*, pair with NLC37 |  |
| NLC38 | CGTTGCAAGACCTGCCTGAA | Located at hygB, forward primer |  |
| Charg4-DR | TTCCCATACGTTTACTCATTCT | Downstream verification reverse primer of *ARG4,* pair with NLC38 |  |
| Charg4-F | CGAGCCAACAAGAACAATG | Forward primer to amplify *ARG4* |  |
| Charg4-R | TGAATCGAATCGCTGACC | Reverse primer to amplify*ARG4* |  |
| qChatg5-F | GCCTCCTGTATGACCTCTACTCTGG | Amplification of *ATG5* in qPCR assays, forward primer | Amplification of *ATG5* expression |
| qChatg5-R | GTTCCTCTTCGCCGCCTTCAAG | Amplification of *ATG5* in qPCR assays, reverse primer |  |
| qChatg7-F | CACGACGGCTCCATCTACGAATG | Amplification of *ATG7* in qPCR assays, forward primer | Amplification of *ATG7* expression |
| qChatg7-R | CGGCATCAACAAGGTAAGTGGTCTC | Amplification of *ATG7* in qPCR assays, reverse primer |  |
| qChatg8-F | ACCCGCCGACTTGACCGTAG | Amplification of *ATG8* in qPCR assays, forward primer | Amplification of *ATG8* expression |
| qChatg8-R | TGGCAGGACCTCATCGACGAAG | Amplification of *ATG8* in qPCR assays, reverse primer |  |
| qChatg9-F | CCGAACACCTCTTCAACCAGTCTC | Amplification of *ATG9* in qPCR assays, forward primer | Amplification of *ATG9* expression |
| qChatg9-R | GCCACGAGTCGCCAAGCATAC | Amplification of *ATG9* in qPCR assays, reverse primer |  |
| qCharg1-F | AGGCTGCGGCTTCGTCTC | Amplification of *ARG1* in qPCR assays, forward primer | Amplification of *ARG1* expression |
| qCharg1-R | TGATGGAGGGCTGTAGTGTGTAG | Amplification of *ARG1* in qPCR assays, reverse primer |  |
| qCharg4-F | CCTACGACCGCCTCTTCTACG | Amplification of *ARG4* in qPCR assays, forward primer | Amplification of *ARG4* expression |
| qCharg4-R | CTCTTCCTTGATCTGTGCCATTCC | Amplification of *ARG4* in qPCR assays, reverse primer |  |
| qChlac1-F | CAGGAGATGCCCGAACAGTTGAG | Amplification of *LAC1* in qPCR assays, forward primer | Amplification of *LAC1* expression |
| qChlac1-R | GTTGACGAGCCATTGGACCAGAC | Amplification of *LAC1* in qPCR assays, reverse primer |  |
| qChlac3-F | CGCCACTGCCAACTACGATGTC | Amplification of *LAC3* in qPCR assays, forward primer | Amplification of *LAC3* expression |
| qChlac3-R | TTGTCAGGAAGAGGAGGTCCAGTC | Amplification of *LAC3* in qPCR assays, reverse primer |  |
| qChbrn1-F | GCCGCATTATCCTCATGGGTTCC | Amplification of *BRN1* in qPCR assays, forward primer | Amplification of *BRN1* expression |
| qChbrn1-R | ACGCAGTTGACAGTGACCTTCTTC | Amplification of *BRN1* in qPCR assays, reverse primer |  |
| qChcmr1-F | TGTTCCGTTGTACTCCTTCCATTGC | Amplification of *CMR1* in qPCR assays, forward primer | Amplification of *CMR1* expression |
| qChcmr1-R | GTCCTCCTTGCTGAAAGTCCGATTC | Amplification of *CMR1* in qPCR assays, reverse primer |  |
| qChpks18F | AGCGTTACGAGGATGCCATTGC | Amplification of *PKS18* in qPCR assays, forward primer | Amplification of *PKS18* expression |
| qChpks18-R | GCGTTGGAGAGGACCTTGTTGTAG | Amplification of *PKS18* in qPCR assays, reverse primer |  |
| BD-cpc1-F | CATATGGCCATGGAGGCCGAATTCATGGGTTGGGCTGGTGGTG | Clone Chcpc1 CDS into pGBKT7 vector | Y2H [analyse](https://fanyi.so.com/?src=onebox#analyse) |
| BD-cpc1-R | TGCGGCCGCTGCAGGTCGACGGATCCTCATTGTCCGCTGTATCCAT |  |  |
| AD-cpc1-F | ATATGGCCATGGAGGCCAGTGAATTCATGGGTTGGGCTGGTGGTG | Clone Chcpc1 CDS into pGBKT7 vector | Y2H [analyse](https://fanyi.so.com/?src=onebox#analyse) |
| AD-cpc1-R1 | GGCGGGATCCAAGGTGTCGGACTGCACAGAGCACAAAGTC |  |  |
| AD-cpc1-R | ATCTGCAGCTCGAGCTCGATGGATCCTCATTGTCCGCTGTATCCAT |  |  |
| AD-cpc1-F1 | CCGACACCTTGGATCCCGCC |  |  |
| AD-atf1-F | ATATGGCCATGGAGGCCAGTGAATTCATGGCAGCTGCATCCCAGACT | Clone Chtup1 CDS into pGADT7 vector | Y2H [analyse](https://fanyi.so.com/?src=onebox#analyse) |
| AD-atf1-R | ATCTGCAGCTCGAGCTCGATGGATCCTCAGTAATTGGAGGGGTAAC |  |  |
| PET32a-atf1-F | CATGGCTGATATCGGATCCATGGCAGCTGCATCCCAG | Clone Chatf1 CDS into PET32a vector | GST-pull down [analyse](https://fanyi.so.com/?src=onebox#analyse) |
| PET32a-atf1-R | GGTGGTGGTGGTGCTCGAGGTAATTGGAGGGGTAACG |  |  |
| PGEX-cpc1-F | TTCCAGGGGCCCCTGGGATCCATGGGTTGGGCTGGTGGTGGTGGT | Clone Chcpc1 CDS into pGEX-6P-1 vector | GST-pull down [analyse](https://fanyi.so.com/?src=onebox#analyse) |
| PGEX-cpc1-R | TCAGTCACGATGCGGCCGCTCGAGTCATTGTCCGCTGTATCCATGAGCCAT |  |  |
| Biotin-pATG5-F | CAGCTATGTTGCGTTAGTAAGTCACCCATCTACGCTGTCGC | Forward primer | EMSA [analyse](https://fanyi.so.com/?src=onebox#analyse) |
| pATG5-F | CAGCTATGTTGCGTTAGTAAGTCACCCATCTACGCTGTCGC |  |  |
| pATG5-R | GCGACAGCGTAGATGGGTGACTTACTAACGCAACATAGCTG | Reverse primer |  |
| Biotin-pATG7-F | TCCCAAATGTCGGTCGTGACCCATCGTTCCCTCCATCTCA | Forward primer | EMSA [analyse](https://fanyi.so.com/?src=onebox#analyse) |
| pATG7-F | TCCCAAATGTCGGTCGTGACCCATCGTTCCCTCCATCTCA |  |  |
| pATG7-R | TGAGATGGAGGGAACGATGGGTCACGACCGACATTTGGGA | Reverse primer |  |
| Biotin-pATG9-F | GCGGCGCGTCACCTGCATGAGTCCCATGTCGATGAGGCGCT | Forward primer | EMSA [analyse](https://fanyi.so.com/?src=onebox#analyse) |
| pATG9-F | GCGGCGCGTCACCTGCATGAGTCCCATGTCGATGAGGCGCT |  |  |
| pATG9-R | AGCGCCTCATCGACATGGGACTCATGCAGGTGACGCGCCGC | Reverse primer |  |
| Biotin-pARG1-F | AAGACAGTGGTGGAGATCAAGTTGGATGAGTAACTTGGCGCTGCACTCGATTCGCGTGTT | Forward primer | EMSA [analyse](https://fanyi.so.com/?src=onebox#analyse) |
| pARG1-F | AAGACAGTGGTGGAGATCAAGTTGGATGAGTAACTTGGCGCTGCACTCGATTCGCGTGTT |  |  |
| pARG1-R | CCAACTTGATCTCCACCACTGTCTT | Reverse primer |  |
| Biotin-pARG4-F | CATTGGCGTTTGGGACTGAATTACAATGAGTAATGAAATAACTCACACCACAAAGCAGTA | Forward primer | EMSA [analyse](https://fanyi.so.com/?src=onebox#analyse) |
| pARG4-F | CATTGGCGTTTGGGACTGAATTACAATGAGTAATGAAATAACTCACACCACAAAGCAGTA |  |  |
| pARG4-R | TACTGCTTTGTGGTGTGAGTTATTTCATTACTCATTGTAATTCAGTCCCAAACGCCAATG | Reverse primer |  |
| Biotin-pATG8-F | GGAGAGCGTGAAGGAGAGAGACGGGATGAGTCAAAGTACCCAGGTAGCCAGGTCATGGGA | Forward primer | EMSA [analyse](https://fanyi.so.com/?src=onebox#analyse) |
| pATG8-F | GGAGAGCGTGAAGGAGAGAGACGGGATGAGTCAAAGTACCCAGGTAGCCAGGTCATGGGA |  |  |
| pATG8-R | TCCCATGACCTGGCTACCTGGGTACTTTGACTCATCCCGTCTCTCTCCTTCACGCTCTCC | Reverse primer |  |
| pABAi-arg1F | AAGCATGGTTAGAAGAAGC | Clone Charg1 CDS into pAbAi vector | Y1H [analyse](https://fanyi.so.com/?src=onebox#analyse) |
| pABAi-arg1R | TATGGAGAAGTGGGGAGT |  |  |
| pABAi-atg8F | TCTTGGAGGACGCAGGTT | Clone Chatg8 CDS into pAbAi vector | Y1H [analyse](https://fanyi.so.com/?src=onebox#analyse) |
| pABAi-atg8R | GGTGGGGATTTGTGAGCGGT |  |  |
| pABAi-arg4F | GGGGTACCTGCATATCAAATGGCTAG | Clone Charg4 CDS into pAbAi vector | Y1H [analyse](https://fanyi.so.com/?src=onebox#analyse) |
| pABAi-arg4-R | CCGCTCGAGCTGAGGTGGTTTCACAAT |  |  |
| Chste7-FP1 | CGAGCGTTTGGCGGTAAT | 5’ flanking region of *STE7,* forward primer | Delete *STE7* |
| Chste7-RP1 | TCCTGTGTGAAATTGTTATCCGCTGGTTGTGGTCGTGTCAGTGTT | 5’ flanking region of *STE7,* reverse primer with hygB gene tail |  |
| Chste7-FP2 | GTCGTGACTGGGAAAACCCTGGCGAAGGGAGTTGCGTGTAGG | 3’ flanking region of *STE7*, forward primer with hygB gene tail |  |
| Chste7-RP2 | CGGATTATGGGAAAGAGG | 3’flanking region of *STE7*, reverse primer |  |
| M13R | AGCGGATAACAATTTCACACAGGA | Forward primer to amplify hygB |  |
| M13F | CGCCAGGGTTTTCCCAGTCACGAC | Reverse primer to amplify hygB |  |
| Chste7-F | AGGCAGAGGATTTGATAGTGT | Forward primer to amplify *STE7* | *STE7* deletion verification |
| Chste7-R | ATATGGTAAACGGTGGACTT | Reverse primer to amplify *STE7* |  |
| Chste11-FP1 | CGAGGATACGAGGGTTGACA | 5’ flanking region of *STE11,* forward primer | Delete *STE11* |
| Chste11-RP1 | TCCTGTGTGAAATTGTTATCCGCTGGTAGATGAGTGCGGTTAGGG | 5’ flanking region of *STE11*, reverse primer with hygB gene tail |  |
| Chste11-FP2 | GTCGTGACTGGGAAAACCCTGGCGATTTCCAGCCTTACATCTT | 3’ flanking region of *STE11*, forward primer with hygB gene tail |  |
| Chste11-RP2 | CATCCCACTACTTAGACCA | 3’flanking region of *STE11*, reverse primer |  |
| M13R | AGCGGATAACAATTTCACACAGGA | Forward primer to amplify hygB |  |
| M13F | CGCCAGGGTTTTCCCAGTCACGAC | Reverse primer to amplify hygB |  |
| Chste11-F | GATGGTGGACGCACTTCA | Forward primer to amplify *STE11* | *STE11* deletion verification |
| Chste11-R | CCGCCAAACGATACATAA | Reverse primer to amplify *STE11* |  |
| Chchk1-FP1 | GCGTGCGAGTAAGCAAGTAA | 5’ flanking region of *CHK1,* forward primer | Delete *CHK1* |
| Chchk1-RP1 | TCCTGTGTGAAATTGTTATCCGCTTGGGAGCAGACGAGTGGAG | 5’ flanking region of *CHK1*, reverse primer with hygB gene tail |  |
| Chchk1-FP2 | GTCGTGACTGGGAAAACCCTGGCGTCTATAAACACCGCCCAATG | 3’ flanking region of *CHK1*, forward primer with hygB gene tail |  |
| Chchk1-RP2 | AGAAATCGCCCGACCTAA | 3’flanking region of *CHK1*, reverse primer |  |
| M13R | AGCGGATAACAATTTCACACAGGA | Forward primer to amplify hygB |  |
| M13F | CGCCAGGGTTTTCCCAGTCACGAC | Reverse primer to amplify hygB |  |
| Chchk1-F | CCGCAACTACGAGACCTT | Forward primer to amplify *CHK1* | *CHK1* deletion verification |
| Chchk1-R | TGGACTCACGCTTCAACT | Reverse primer to amplify *CHK1* |  |
| BD-chk1-F | GGAATTCATGCCACCAGCAGGAAGCGG | Clone Chchk1 CDS into pGBKT7 vector | Y2H [analyse](https://fanyi.so.com/?src=onebox#analyse) |
| BD-chk1-R | CGGGATCCTTATCGCATAATCTCCTGGT |  |  |
| pET32a-ste7-F | CATGGCTGATATCGGATCCATGGCAGAACCGAATTTCAAA | Clone Chste7 CDS into PET32a vector | In vitro phosphorylation assay |
| pET32a-ste7-F1 | GGGAGACATTGCTGAGGACTTCGTAGGAACTGGTACATAC |  |  |
| pET32a-ste7-R1 | ACGAAGTCCTCAGCAATGTCTCCCTCCAGCTCACTCGATACACC |  |  |
| pET32a-ste7-R | GGTGGTGGTGGTGCTCGAGTTGAACTTCGTTGTTCCACCG |  |  |
| pET32a-cpc1-F | CATGGCTGATATCGGATCCATGGGTTGGGCTGGTGGT | Clone Chcpc1 CDS into PET32a vector | GST-pull down [analyse](https://fanyi.so.com/?src=onebox#analyse) |
| pET32a-cpc1-R | GGTGGTGGTGGTGCTCGAGTTGTCCGCTGTATCCATGAGC | Clone Chcpc1 CDS into PET32a vector |  |
| pET32a-chk1-F | CATGGCTGATATCGGATCCATGGAGACGGACATGCATCGC | Clone Chchk1 CDS into PET32a vector | In vitro phosphorylation assay |
| pET32a-chk1-R | GGTGGTGGTGGTGCTCGAGGGTTGTCCTTGTTCTTGTCAA | Clone Chchk1 CDS into PET32a vector |  |
| qChchk1-F | GGCAAGGACTACCACCACCAG | Amplification of *CHK1* in qPCR assays, forward primer | Amplification of *CHK1* expression |
| qChchk1-R | AGGGCAGAGATCGGATGTATTCG | Amplification of *CHK1* in qPCR assays, reverse primer |  |
| qChste7-F | GGCTGGCGATGCTCAAATACCC | Amplification of *STE7* in qPCR assays, forward primer | Amplification of *STE7* expression |
| qChste7-R | GCTGACTGTTCCTCCGTTTCCTG | Amplification of *STE7* in qPCR assays, reverse primer |  |
| qChste11-F | AAACAGGCAATCGCTCGCTACC | Amplification of *STE11* in qPCR assays, forward primer | Amplification of *STE11* expression |
| qChste11-R | CGCTTCTCCGTCCGTGAACTTATAG | Amplification of *STE11* in qPCR assays, reverse primer |  |
